# A review of *Psoralea corylifolia* L.: a valuable plant with profound biological significance

**DOI:** 10.3389/fphar.2024.1521040

**Published:** 2025-01-20

**Authors:** Anni Yang, Lingping Kong, Zhibo You, Xinyu Li, Jian Guan, Fengjin Li, Lingyun Zhong, Hai Jiang

**Affiliations:** ^1^ Key Laboratory of Basic and Application Research of Beiyao, Heilongjiang University of Chinese Medicine, Ministry of Education, Harbin, China; ^2^ Division of Gastroenterology, Institute of Digestive Disease, Qingyuan People’s Hospital, The Affiliated Qingyuan Hospital of Guangzhou Medical University, Qingyuan, Guangdong, China; ^3^ School of pharmacy, Jiangxi University of Chinese Medicine, Nanchang, China

**Keywords:** *Psoralea corylifolia* L., ethnobotany, phytochemistry, pharmacological effects, hepatotoxicity

## Abstract

*Psoralea corylifolia* L. (PCL) is an annual herb of the genus Psoralea in the family Fabaceae, and its mature fruit can be used medicinally as a precious medicinal herb to tonify muscles and bones. With the deepening of research, its applications to various industries, including food, agriculture, and cosmetics, with products being developed in countries such as Vietnam, India, and Japan. A total of 321 metabolites, including coumarins, flavonoids, meroterpenes, benzofurans, and dimers, were identified in PCL. PCL and related products have demonstrated therapeutic effects, such as antiosteoporosis effects, estrogen-like effects, anti-inflammatory properties, neuroprotection, antitumor activity, and vitiligo treatment. The expression mechanisms of these pharmacological effects are closely related to the regulation of the immune system, the inhibition of oxidative stress, and the induction of apoptosis. This paper summarizes the latest research on the ethnobotany, phytochemistry, processing technology, pharmacology, and hepatotoxicity of PCL. Furthermore, bibliometric analysis was used to systematically analyze the research hotspots and trends in PCL, which have never been addressed in previous reviews of PCL. In the future, it will be necessary to focus on the active metabolites of PCL, analyze its targets and signaling pathway network to address potential toxicity and side effects in clinical applications, and further expand the potential application of PCL in medicine.

## 1 Introduction

**FIGURE 1:**
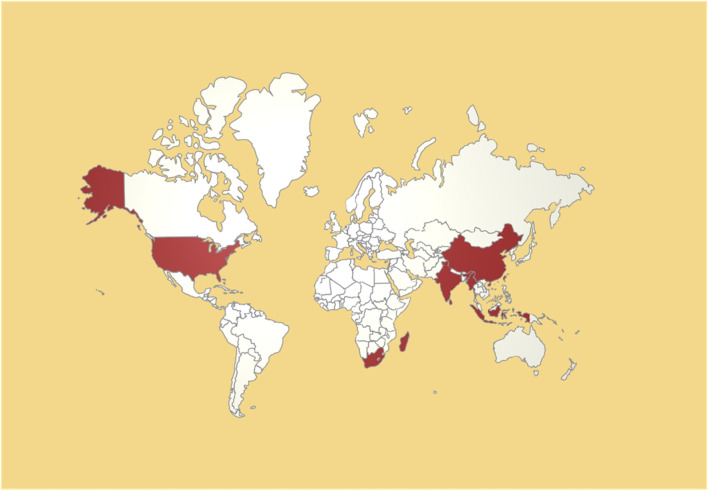
World map showing the distribution of *Psoralea corylifolia* L. (red).

*Psoralea corylifolia* L. (PCL) belongs to the genus Psoralea in the Fabaceae family and is known as Hujiuzi or Poguzhi. Notably, the genus is named after the Greek word psoraleos, which means “suffering from itching or leprosy” (Chopra et al., 2013). The plant is well suited for thriving in warm, humid, and sunny climates, primarily in tropical and subtropical regions such as Southeast Asia, India, and southern Africa (Khushboo et al., 2010). It is a commonly used spice and medicinal plant in the region and is often used in flavorings and pharmaceuticals (Khushboo et al., 2010). China is a significant PCL producer and is concentrated mainly in provinces such as Yunnan, Sichuan, Hebei, Shanxi, Anhui, Jiangxi, Henan, Guangdong, Guangxi, and Guizhou. The cultivation of PCL in China is predominantly either cultivated or wild (Editor’s Committee of Chinese Flora, China Academy of Sciences, 2006). The distribution of PCL worldwide is shown in Figure 1.

In many research and application scenarios, not only the application of different botanical drugs of PCL, but also the related formulations from PCL and many different types of extract forms are covered. In China, the dried and mature fruit of PCL was recorded as a Chinese herbal medicine in the China Pharmacopoeia and named Psoraleae Fructus (PF). PF is warm in nature and is effective at tonifying the kidney, increasing yang energy, warming the spleen, alleviating diarrhea, and relieving asthma (Compilation of National Pharmacopoeia Committee, 2020). It is a highly respected medicinal material in traditional Chinese medicine (TCM) and has been actively explored and applied in the prevention and treatment of many diseases. In traditional Indian therapies, the whole plant of PCL is used to treat vitiligo and infectious skin diseases (Gidwani et al., 2010). Modern medicine has further explored the role of PCL extract in regulating skin pigment metabolism on the basis of traditional medication experience has led to the development of ointments containing powdered seeds of PCL for vitiligo treatment (Hussain et al., 2016). Moreover, a compound preparation made from PCL has also been used to treat orthopedic diseases (Huang, 1984). The plant has been shown to have antioxidant activity in Classified Materia Medica (Tang and Cao, 1991), which is believed to delay aging and strengthen the body’s resistance, and has been made into Qing’e pills. Currently, the focus of research has shifted to exploring the protective effect of PCL extract against oxidative stress in nerve cells to prevent and treat neurodegenerative diseases (Guo et al., 2024). Although there has been no clear record of anticancer application in the past, active metabolites in PCL have demonstrated potential anticancer value in a variety of *in vitro* cellular experiments and animal model experiments on various types of cancers (Liu et al., 2024). Furthermore, the estrogenic effects of PCL extract offer a new option for women’s healthcare (Li and Song, 2019). Phytochemical studies have revealed that PCL contains a variety of beneficial metabolites, such as flavonoids, coumarins, meroterpenes, and benzofurans (Yang et al., 2024), which exhibit potent biological activities.

In recent years, the use of PCL has expanded into various fields, including domestic cosmetics and agricultural disease prevention. Bakuchiol is highly valued for its skincare benefits, such as anti-aging, anti-pigmentation, and anti-acne properties (Mascarenhas-Melo et al., 2024), and is now a key ingredient in daily skincare products such as creams and sunscreens (Lau et al., 2010; Liu, 2023). In agricultural disease control, PCL extract has been shown to be effective against plant pathogens such as apple canker, cucumber anthracnose, wheat scab, and rice blast (Guan et al., 2007). It has reduced the use of chemical pesticides, ensuring the safety of agricultural products and the health of the ecological environment.

PCL is crucial in various industries, such as medicine, beauty, and agriculture. In recent years, most reviews on PCL have focused on its pharmacological properties or phytochemical extraction and isolation (Chen L. et al., 2023). In addition to providing a more comprehensive introduction to the phytochemistry of PCL, this paper analyzes the research hotspots and trends of PCL with the help of bibliometrics. This is the first time that this analytical perspective has been applied in previous reviews on PCL, and it is innovative. In addition, this paper summarizes the current status of the application of PCL in the world, and includes more application examples and practical experiences in different regions, which is useful reference for the further development of PCL in China. Moreover, this paper provides a detailed review of the various known methods for reducing the toxicity of PCL, which is of great significance for effectively reducing the potential risks in the application of PCL and guaranteeing its safety. We hope that this review fully explores and further enhances the comprehensive value of PCL and contributes to human health.

## 2 Methodology

This review searched the literature on PCL in the CNKI, VIP, Wanfang and Web of Science Core Collection databases. All the databases were searched with advanced search tools, and the keyword “*Psoralea corylifolia* L.” was used to ensure the accuracy of the search topic. The publication period was from 1 January 2000, to 31 October 2024, and the data were further screened and cleaned. Further data screening and cleaning were performed, including manually removing duplicates in the database, removing documents with missing authors or keywords, deleting documents with poor relevance or incomplete data, and distinguishing between English-language documents published by Chinese authors and English-language documents published by foreign authors. The finalized sample data included 716 Chinese and 510 English documents. After duplicates were effectively eliminated, CiteSpace was used to analyze and visualize the data, and “YearsPerSlice” was set at 1 year to ensure the timeliness and meticulousness of the analysis. In “NodeTypes,” the selected node types include “Author,” “Country,” “Institution,” and “Keyword” to build a knowledge network map covering key research participants and topics. All other settings used the software’s system defaults to ensure the consistency and reproducibility of the analysis.

Supplementary Figure 1A illustrates the keyword time zones in the field of PCL research. In the early 2000s, research focused on extracting and isolating new chemical metabolites. However, since 2017, there has been a noticeable shift toward research focused on exploring the potential mechanisms of metabonomics and signaling pathways. In recent years, concerns have arisen regarding potential adverse reactions associated with PCL in clinical settings, particularly the increasing incidence of liver injury. The geographic visualization of the number of PCL articles in Supplementary Figure 1B shows that China holds a prominent position in PCL research, demonstrating significant academic influence and discourse power. India, Japan, and the United States follow closely behind, actively contributing to high-quality scientific research in the PCL field.

## 3 Botany

**FIGURE 2:**
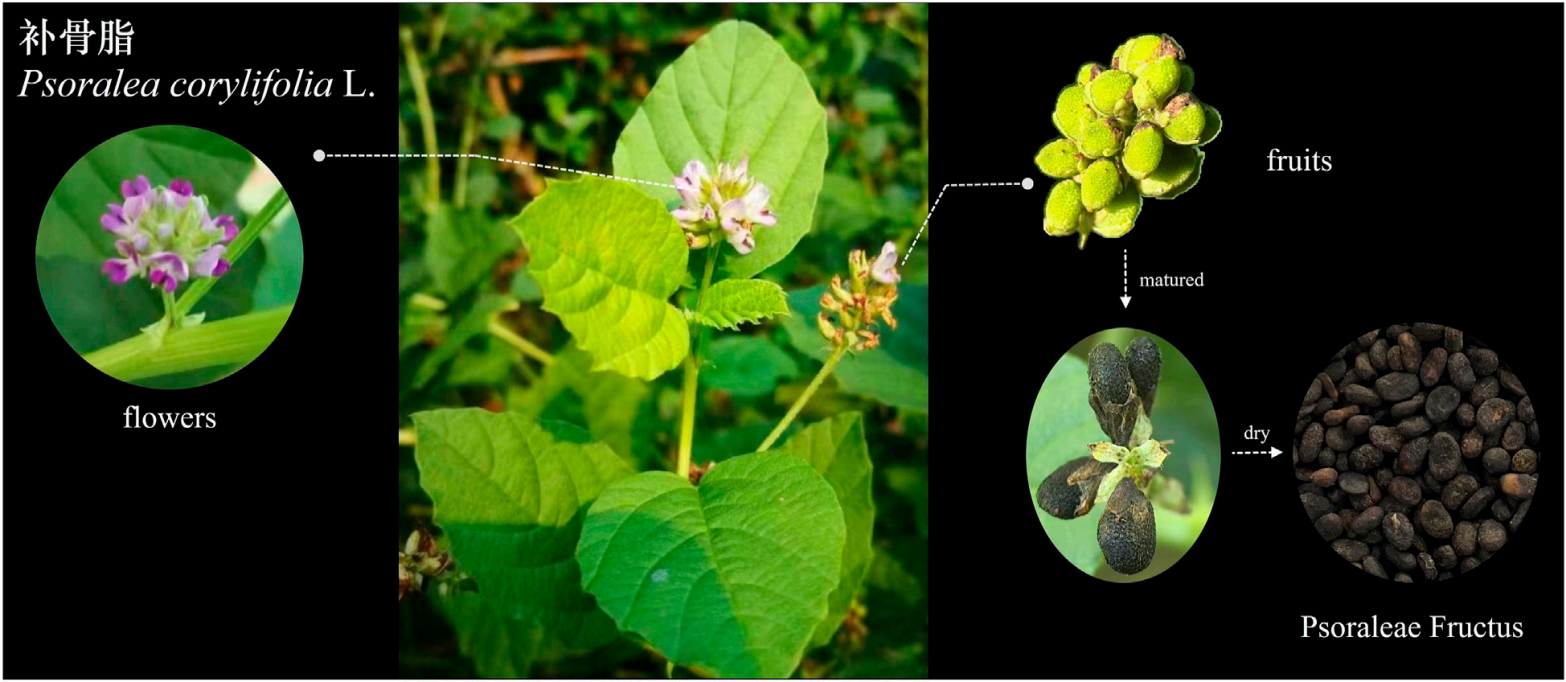
Morphological characteristics of the leaves, flowers and fruits of *Psoralea corylifolia* L.

PCL is a unique plant with distinct features in its leaves, inflorescences, and fruits. It is a tall annual botanical herb that can reach heights of 60–150 cm. The leaves are simple and oval shaped, with thick serrated edges and black glandular patches on both sides. The flowers are white and glandular and are arranged in dense racemes or small heads with 10–30 flowers. The fruits are oblate pods that are approximately 5 mm long and black (Editor’s Committee of Chinese Flora, China Academy of Sciences, 2006). PF, which represents the dried and mature fruit of PCL, is harvested during the autumn season and has medicinal value. PF is oblate or kidney-shaped, with a rough surface and a slightly concave center. The inside is a kernel with two cotyledons rich in oil, ranging from light brown to yellowish brown (Compilation of National Pharmacopoeia Committee, 2020). The morphology of the entire plant, including its leaves, flowers, and fruits, is illustrated in Figure 2.

## 4 Ethnobotany

### 4.1 Usage in traditional Chinese medicine

PCL, also known as “Hujiuzi,” is believed to have originated in India and was introduced to China during the Tang Dynasty (Li and Zhu, 2017). As time passed, the documentation of PCL in medical texts from various dynasties became more extensive. The “Yi Lin Cuan Yao” briefly mentioned the ability of PCL to treat cold and asthma deficiencies. Moreover, the “Theory of Medicinal Properties” (Zhen, 1983) provides more detail about its benefits for men’s lumbago, knee joint chills, and chronic gastroenteritis. Further research in “Kaibao Materia Medica” (Lu and Shang, 1998) detailed the medicinal effects of PCL in improving deficiency and cold symptoms, enhancing men’s kidney yang, and regulating women’s blood qi. Subsequent medical texts, such as the “Compendium of Materia Medica” and the “Medicinal Solution of Sorbus fragrans” (Huang, 2017), continued to expand on the PCL’s efficacy and evaluation, emphasizing its role in restoring vital energy and strengthening the body. The historical use of PCL in TCM reflects its importance and provides a strong foundation for its modern application.

The pharmacological effects documented in ancient texts lay the groundwork for the clinical application of PCL and the evolution of Chinese patent medicine. It is commonly used with fennel and other medications to address symptoms such as kidney Yang deficiency, impotence, infertility, and waist and knee cold pain, as seen in the classic Buguzhi Pills (Li K. et al., 2019). To treat asthma caused by kidney deficiency, psoralen can be combined with medications such as cinnamon, which are well known as HeiXi Dan (Guan, 1955). For diarrhea caused by spleen and kidney yang deficiency, PCL is often used with Euodiae Fructus, Schisandrae chinensis Fructus, and nutmeg in formulations such as Sishen Pill (Zhang et al., 2019).

### 4.2 Usage in other countries

PCL occupies an essential position in TCM and is widely used in China. Its influence goes far beyond that and is also highly regarded globally. In India, it is referred to as “Kushtanashini,” and locals believe that every part of the plant can treat various skin conditions such as vitiligo, rashes, and infections (Khushboo et al., 2010). In Vietnam, the alcohol extract of PCL is used to alleviate rheumatism (Editor’s Committee of Chinese Flora, China Academy of Sciences, 2006). In addition to their medicinal uses, PCL seeds are also used to produce sesame oil (Khushboo et al., 2010). In Japan, the alcohol extract of PCL is utilized as a food preservative in processed foods and pickles (Qiao et al., 2007), highlighting its potential in the food industry. Table 1 provides an overview of the traditional uses of PCL documented globally.

**TABLE 1:** World records of traditional applications of *Psoralea corylifolia* L.

Country	Ethnic	Ethnic medicine names	Plant parts	Use		Reference
				Internal use	External use	
China	Mongolian	Ximodula	Fruits	Used for cold pain in waist and knees, frequent urination, enuresis and diarrhea in the elderly	Topical treatment of vitiligo, corn, psoriasis and alopecia	Jia and Li (2005)
	Bai	Heizizi	Seeds	Waist and knee pain, senile enuresis, diarrhea, nocturnal emission, sexual neurasthenia	Vitiligo, corns, psoriasis, baldness	
	Jingpo	wuijvangchi		Waist and knee pain, senile enuresis, neurasthenia	Topical treatment of vitiligo and psoriasis	
	Deang	Psoralea corylifolia/Psoraleae Fructus				
	Achang	Mendeerguwu				
Vietnam			Fruits		Rheumatism	Editor’s Committee of Chinese Flora, China Academy of Sciences (2006)
India		Kushtanashini	Roots, stems, leaves, seeds, flowers	Leprosy, asthma, nephritis, osteoporosis, impotence, gynecological bleeding	Vitiligo, insect repellent, alopecia areata and alopecia	Khushboo et al. (2010)
Japan		Psoralea corylifolia/Psoraleae Fructus	Fruits	As a food additive		Qiao et al. (2007)

## 5 Phytochemistry

The study of PCL has a lengthy history, tracing back almost a century. Contemporary research has focused predominantly on coumarins, flavonoids, meroterpenes, benzofurans, and dimers in the whole plant, all of which have exhibited encouraging pharmacological effects in combating inflammation, oxidation, and cancer (Lu et al., 2019). Supplementary Table 1 provides a comprehensive compilation of the chemical metabolites discovered in PCL, and the molecular structures of these metabolites are visually depicted in Supplementary Figure 2.

### 5.1 Coumarins

Coumarin metabolites are critical active metabolites found in PCL and consist primarily of two structural central cores: furocoumarin and coumestrol. Furan coumarins are a group of metabolites that form furan rings through the condensation of the 7-hydroxyl group of the benzo α-pyranone central core with the 6- or 8-substituted isopentenyl group (Kuang, 2003). Research on coumarin metabolites began in 1933 (Wei et al., 2019). Psoralen (1) and isopsoralen (2) are prominent examples of these metabolites and are listed in the China Pharmacopoeia (2020 edition) for content determination in PCL medicinal materials. Additionally, estrogenic metabolites have coumestrol as the central core structure, another active metabolite in PCL (Ji and Xu, 1995). These metabolites exhibit estrogen-like activities, bind to estrogen receptors and activate related signaling pathways, showing significant pharmacological effects such as antiosteoporosis (Cai et al., 2021), anti-inflammatory, neuroprotective (Li et al., 2017b), and antitumor activities (Sharifi-Rad et al., 2020). In the 20th century, research on estrogenic metabolites in PCL made a breakthrough, with Xu et al. successfully identifying six new estrogenic metabolites (28–33) (Xu Q. et al., 2022). Currently, 36 coumarins have been isolated and identified from PCL, and their chemical structures are depicted in Supplementary Figure 2A.

### 5.2 Flavonoids

Flavonoids are derived from benzopyranone or benzopyranol and are commonly found in numerous plants, playing crucial roles as effective metabolites in PCL (Kuang, 2003). PCL contains a variety of flavonoids, with the main structural types being flavonoids, flavonols, dihydroflavonoids, isoflavones, chalcones, flavanols, and aurones (Yang et al., 2024). More information can be found in Supplementary Figures 2B–E. Flavonoids and flavonol metabolites are not very abundant in PCL. Astragalin (37) was first identified in 1995 (Ji and Xu, 1995), followed by the discovery of six related metabolites (38–43). Dihydroflavonoids are produced through the hydrogenation of double bonds at the 2nd and 3rd positions in the basic structure of flavonoids. The earliest research on dihydroflavonoids in PCL dates back to the isolation of bavachin (44), bavachinin (45), and isobavachin (46) in 1968 (Bhalla et al., 1968). Since then, various dihydroflavones (47–79) have been isolated. Isoflavones, with a central core structure of 3-phenylchromone, are the most commonly reported metabolites in PCL. 57 isoflavones (80–136) have been extracted from PCL. Among these, 3 isoflavones (134–136) were recently discovered by Shi (Xu Q.-X. et al., 2023) in 2023, demonstrating anti-proliferation and anti-metastasis effects against human non-small cell lung cancer. Chalcone, a derivative of the dihydroflavone C-ring formed by breaking the 1- and 2-position bonds, is a ring-opening metabolite. Bavachalcone (137) and isobavachalcone (138) were initially identified in 1968, followed by the identification of 33 related metabolites (139–171). Flavanol metabolites were not reported until recently, with bavaisoflavanol (172) being isolated from PCL in 2022 (Xu Q. et al., 2022). Another significant discovery occurred in 2024 when 6-(γ,γ-dimethylallyl)-4′-hydroxy-7-methoxy-(2R,3R)-dihydroflavonol (173) was found in PCL. Although rare, 12 different aurone metabolites (174–185) have also been extracted from PCL. Flavonoids possess remarkable biological activities that make them promising for preventing and treating various diseases. Given their abundance in PCL, flavonoids are anticipated to serve as crucial metabolites for evaluating PCL quality in the future.

### 5.3 Meroterpenes

Meroterpenes are metabolites produced through the combination of the polyisoprene pathway (terpene) and other biosynthetic pathways (non-terpene metabolites) (Nazir et al., 2021). They are a subset of terpenoids with complex and diverse chemical structures that have attracted significant attention from researchers. PCL is particularly rich in meroterpenes, with a range of 59 different metabolites identified. One notable meroterpene found in PCL is bakuchiol (186), which has been shown to possess antioxidant, anti-inflammatory, and antioxidative properties (Puyana et al., 2022). Bakuchiol has even been incorporated into skincare products because of its beneficial effects (Feng, 2023). More recently, researchers have discovered that meroterpenes in PCL can also exist as dimers, such as bisbakuchiol A-U (245–266), which exhibit promising antiviral, antibacterial (Yang et al., 2022), anti-inflammatory, and antitumor activities (Xu et al., 2021).

### 5.4 Benzofurans

Benzofurans are essential heterocyclic metabolites. In 1992, two benzofurans, corylifonol (267) and isocorylifonol (268), were isolated from PCL (Kuo and Lin, 1992). Seven benzofurans have been identified (He et al., 2021), including dihydrobutylcnideoside A (269) and butylcnideoside A (270), as well as methylcnidioside A (271), isopsoralenoside butyl ester (272), isopsoralenoside methyl ester (273), dihydroisopsoralenoside methyl ester (274), and dihydroisopsoralenoside methyl ester (275). The chemical structures of these metabolites are depicted in Supplementary Figure 2H.

### 5.5 Dimers

Dimers are molecules of two identical or different molecules linked by chemical bonds. In addition to the homodimers of heteroterpenes (245–266) found in PCL, 17 heteroterpene heterodimers known as psocorylins A-Q (276–292) were also discovered (Xu et al., 2020), and their chemical structures are depicted in Supplementary Figure 2I. These metabolites involve the flavonoids psocorylins A-Q (276-290, 292) and coumarin psocorylins P (291) as the unit structures responsible for polymerization. The two monomer units of these metabolites are connected primarily by C-C or C-O-C bonds. Furthermore, in 2022, nine new flavonoid dimers known as psocorylins R-Z (293–301) were isolated from PCL (Xu Q.-X. et al., 2023). The dimerization of these monomer structures enhances the complexity and diversity of both their structure and biological activity.

### 5.6 Others

PCL also contains trace elements, lipids, glycosides, volatile oils, and various other metabolites. Some of the chemical structures of these metabolites are depicted in Supplementary Figure 2J. Among these elements are potassium, manganese, calcium, iron, copper, zinc, arsenic, antimony, rubidium, strontium, selenium, and others (Guo et al., 2006), which are all essential for sustaining life processes. Lipid metabolites found in PCL include monoglycerides, diglycerides, triglycerides, free fatty acids, phospholipids, hydrocarbons, wax fats, and polar lipids (Wei et al., 2019). Glycosides such as glucose, raffinose, carotene, methyl glycoside, PCL polysaccharide, and β-sitosterol-D-glucoside can regulate human metabolism and increase immunity (Lu et al., 2019). The volatile oils and fatty acids in PCL, such as palmitic acid, oleic acid, linoleic acid, stearic acid, linolenic acid, and tetracarboxylic acid, have nutritional and health benefits and serve as essential metabolites in pharmaceutical preparations (Qiu et al., 2010). While these metabolites may not directly produce pharmacological effects, they are crucial for maintaining normal metabolism.

PCL contains numerous valuable active metabolites that form the basis for its pharmacological effects. Nevertheless, the original plant contains low levels of these active metabolites, and their complex structure complicates the extraction and separation process. Furthermore, it is important to continue isolating additional potential active metabolites from PCL and utilize the current research findings to establish a molecular library of metabolites.

## 6 Processing technology

Processing, as a unique Chinese technology, occupies an extremely critical position in the field of Chinese medicine applications. Under the normative guidance of the Chinese Pharmacopoeia, PF is selected for processing. The properties of PF can be altered by processing to reduce its toxicity or side effects while enhancing its efficacy.

### 6.1 Process history

The processing of PF via various methods and detailed standards has a long history. The earliest known method of processing PF involved soaking and steaming with wine, as described in “Lei’s Treatise on Preparing Drugs.” Over time, techniques such as frying with honey, steaming with wine, and frying with salt were developed during the Northern and Southern Dynasties. The Song Dynasty introduced washing, cutting, vinegar, and medicinal juice processing technologies. During the Ming dynasty, additional methods such as bread, bran, salt, and wine were introduced. The processing of PF has evolved over the centuries, with each period contributing innovations to improve the quality and effectiveness of the final product. During the Qing Dynasty, advanced processing techniques emerged, such as flour frying, bran frying, milk making, and walnut oil production (Liu et al., 2022). Salted PF is included in the China Pharmacopoeia and has been extensively documented in herbal monographs from the Southern and Northern Dynasties to the Qing Dynasty.

At present, traditional processing methods such as salt processing and frying dominate the processing of PF. Other processing methods may diminish due to technical complexities, lack of cost-effectiveness, or failure to meet modern pharmaceutical standards. However, we should recognize the wisdom and value of ancient processing, by enhancing these techniques with modern scientific and technological advancements, new insights and approaches may be discovered, expanding the potential applications and therapeutic benefits of PF.

### 6.2 Effects of processing on the chemical composition

During medicinal material processing, metabolites undergo biological transformation due to auxiliary materials and elevated temperatures, leading to a modification of their pharmacological effects. Psoralen and isopsoralen are the main coumarins present in PF. The levels of psoralen and isopsoralen increase after processing via various methods. Psoralenoside and isopsoralenoside are converted into psoralen and isopsoralen by β-glucosidase (Wang H. et al., 2021). In general, the total amount of coumarin metabolites steadily increased following processing via the Leigong method, the wine-soaking method, the stir-frying method, and the China Pharmacopoeia (Wang et al., 2023a).

There are several processing techniques for PF, and the impacts of these methods on flavonoids exhibit diverse trends. The content was greatly increased through soaking and frying via the wine and pharmacopeia methods, whereas it was notably reduced through frying and the Leigong method (Li et al., 2017a). The total flavonoid concentration of PF slightly increased after salting, possibly because PF is a seed medicinal material. The seed coat is broken during salting, allowing its metabolites to dissolve (Yan et al., 2013). Tao et al. reported that the level of 4′-O-methyl psoralen in three different processed products, fried, salt-baked, and wine-baked, notably increased (Tao et al., 2016). Conversely, the amount of flavonoid methyl ether in PF decreased significantly, likely due to the breakdown and alteration of flavonoid methyl ether in PF at high temperatures. Wang et al. reported a notable increase in the concentrations of bavachin, corylin, isobavachalcone, and bavachalcone in salt-baked items, alongside a sharp decrease in the contents of bavachromanol and bakuchiol (Wang et al., 2014). The variation in the results could be attributed to the combined impact of temperature, time, auxiliary materials, and other factors involved in processing.

After processing, the levels of psoralen in monoterpenoids decreased, likely due to its volatile nature, which caused a decrease in content at high temperatures and its conversion into psoralen and isopsoralen through enzyme activation *in vivo* (Wang et al., 2023a). Additionally, methods such as stir-frying, oil frying, salt frying, salt steaming, and the Leigong method were found to decrease the proportion of monoterpenes, increase the concentration of sesquiterpenes, and diversify the range of volatile metabolites present (Li H. et al., 2021).

The presence of trace elements also impacts the biological activity of PF. Following the application of the Leigong method, Guo et al. observed an increase in the concentrations of six trace elements - Cu, Mn, Ca, Mg, Fe, and Zn - linked to the “kidney,” thereby increasing the efficacy of PF (Guo et al., 2006). This may be due to the loosening of the tissue structure of PF during processing, resulting in an increased rate of dissolution of the metabolites mentioned above and a transformation of the metabolites in PF.

Currently, most studies focus on comparing the metabolite changes in PF before and after processing. The change of one or several index metabolites can only partially reveal the change law of chemical substances *in vitro* during processing. Therefore, enhancing the pharmacological research on the effects of PF *in vivo*, as well as monitoring the dynamic changes *in vivo* through continuous and multilevel monitoring is essential.

### 6.3 Effects of processing on pharmacological action

Studies have shown that salt-roasted PF can effectively treat diarrhoea caused by spleen deficiency. Zhou et al. discovered that after salt roasting, the absorption of psoralen and isopsoralen in the body increased while metabolism decreased, potentially enhancing the anti-diarrheal effects (Zhou et al., 2022). Wu et al. noted that salt processing may enhance the extraction of flavonoids from the Buguzhi pill, a process closely linked to its anti-diarrheal properties (Wu et al., 2021). However, the absorption and distribution process of Buguzhi pills *in vivo* still needs to be explored, despite the examination of the *in vitro* dissolution of chemical metabolites.

According to the traditional theory of “salt roasting into the kidney” in TCM, PF is often roasted with salt for medicinal use in treating osteoporosis (Hou et al., 2023). Following salt roasting, PF was found to inhibit uterine atrophy in ovariectomized rats, increase serum Ca and P levels; decrease ALP, OC, and E2 levels; significantly improve BMD and bone biomechanical strength; and enhance the structural integrity of trabecular bone (Xu Y. et al., 2022). The enhancement of effective metabolites such as psoralen and isopsoralen through salt roasting increases their presence in the bloodstream. Additional investigations are necessary to validate the target and pathway involved. The medicated serum of salt-roasted PF significantly enhances the proliferation and differentiation of osteoblasts, surpassing the effects of raw PF (Gao et al., 2015). Feng and Hu revealed that the absorption rate of salt-baked products was significantly greater than that of raw PF, shedding light on the impact of processing on drugs from a pharmacokinetic perspective (Feng and Hu, 2009). Yan and Gao conducted a thorough study on how various processing methods impact the absorption of coumarins, flavonoids, and monoterpenoids in the intestine of PF (Yan and Gao, 2020). These findings revealed that salt roasting can simultaneously increase the absorption of all three metabolites in the intestine, thereby supporting its potential for improved treatment of osteoporosis.

In addition to enhancing the antiosteoporosis effect of processed PF, a preliminary comparison and analysis were conducted on its antibacterial, antioxidant, and hypoglycemic activities. Li et al. studied the antibacterial properties of raw and processed PF products against *Staphylococcus aureus*, methicillin-resistant *S. aureus*, and extended spectrum β-lactamase *S. aureus* (Li et al., 2012). The variation in the wine-roasted significantly enhanced the antibacterial activity. However, further research is needed to compare the different metabolites in raw and wine-roasted products and explore how these metabolites affect antibacterial activity.

Different portions of raw and processed PF exhibited different levels of antioxidant activity, with significant variations observed among the different processing techniques (Zhang et al., 2013a). Both raw and processed PF demonstrate a more potent α-glucosidase inhibitory effect than dose acarbose (Zhang et al., 2013b). This study demonstrated the impact of processing on different pharmacological effects of PF to some extent. However, these results are primarily based on *in vitro* experiments, highlighting the need for more *in vivo* studies to confirm and further investigate the alterations and mechanisms of these pharmacological activities in a setting closer to the actual physiological environment.

Currently, research is focused on improving the effectiveness of PF through processing, particularly in exploring the benefits of its salt-roasted products for treating osteoporosis. However, these studies focused primarily on changes in the metabolites of the active substances after processing, with limited discussion on the specific mechanisms and targets of the drug’s action.

### 6.4 Effects of processing on hepatotoxicity

#### 6.4.1 Leigong processing method

During Leigong processing, hepatotoxic metabolites are leached out through soaking and bleaching in wine. Steaming then transforms any remaining hepatotoxic metabolites into active metabolites, ensuring that the content of (iso)-psoralen remains within a safe and effective range (Li Z. et al., 2021). This process effectively reduces hepatotoxicity in the final product.

Research has shown that the toxic elements of PF are mostly fat soluble and that the ethanol extract of PF is more hepatotoxic than the water extract is Chen Y. et al. (2017). Song et al. utilized 3D-cultured human liver-like organs in conjunction with high-content imaging technology to assess the impact of the Leigong method (Song et al., 2020). These findings revealed that under optimal experimental conditions, there was no hepatocyte toxicity at the four different concentrations. Interestingly, the higher the alcohol concentration is, the greater the attenuation effect, offering valuable insights into mitigating the risk of liver injury from PF.

The hepatotoxicity of PF notably decreased after the Leigong method was applied, possibly due to modifications in chemical metabolites before and after processing. Through Leigong processing, Lv et al. noticed that the hepatotoxic metabolite (iso)-psoralenoside found in PF was transformed into the active metabolite (iso)-psoralen (Lv et al., 2020). Li et al. observed a significant reduction in the levels of psoralen and isopsoralen after rinsing with running water and soaking in cold water (Li Z. et al., 2021). Hong et al. combined the salt-roasted method with the Leigong method and assessed the changes in hepatotoxicity before and after processing (Hong et al., 2021). The results revealed a significant decrease in hepatotoxic metabolites in processed PF, leading to a notable reduction in hepatotoxicity in mice.

#### 6.4.2 Salt-roasted methods

Salt roasting can reduce the impact on major organs such as the liver, spleen, kidney, thymus, and stomach, thereby reducing hepatotoxicity. It can also decrease the effects on serum cAMP/cGMP, TNF-α, and Na^+^-K^+^-ATPase to alleviate dryness (Xia et al., 2016). Liang et al. verified that PF can improve medication side effects on liver and kidney function in rats with renal-yang insufficiency while also decreasing dryness by regulating AQP gene expression *in vivo* (Liang et al., 2017). Some studies have shown that salt-roasted PF may still have hepatotoxic effects, impacting its safety for clinical use. Fu et al. discovered that salt-roasted PF was more likely to cause liver damage than raw PF was (Fu et al., 2022). In the group that received salt-roasted PF, there was a more significant increase in the IL-6 level in the blood and a more pronounced decrease in the FXR and SULT1E1 levels in the liver. Psoralen, isopsoralen, and corylifolia were identified as potentially effective metabolites of PF and primary hepatotoxic metabolites. Both the water and alcohol extracts of salt-roasted PF were cytotoxic to HK-2 cells, Hep-G2 cells, and LLC-PK1 cells, with the alcohol extract being more hepatotoxic (Wang et al., 2023b). Cai et al. suggested that the presence of Na^+^ in salt-prepared PF altered the intestinal osmotic pressure and renal absorption function in mice, leading to increased absorption and reabsorption of all PF metabolites, thereby enhancing their hepatotoxic effects (Cai et al., 2017). While processing PF can reduce its hepatotoxicity, it cannot eliminate it. Therefore, caution should be exercised when using PF to avoid excessive or prolonged use.

Research on liver injury caused by processed Chinese medicine is still in its early stages, with a focus on evaluating changes in hepatotoxic metabolites. However, studies on metabolic differences *in vivo* before and after processing are lacking. Balancing the attenuation of hepatotoxicity with maintaining efficacy is a challenge that needs to be addressed in future research.

## 7 Pharmacology

**FIGURE 3:**
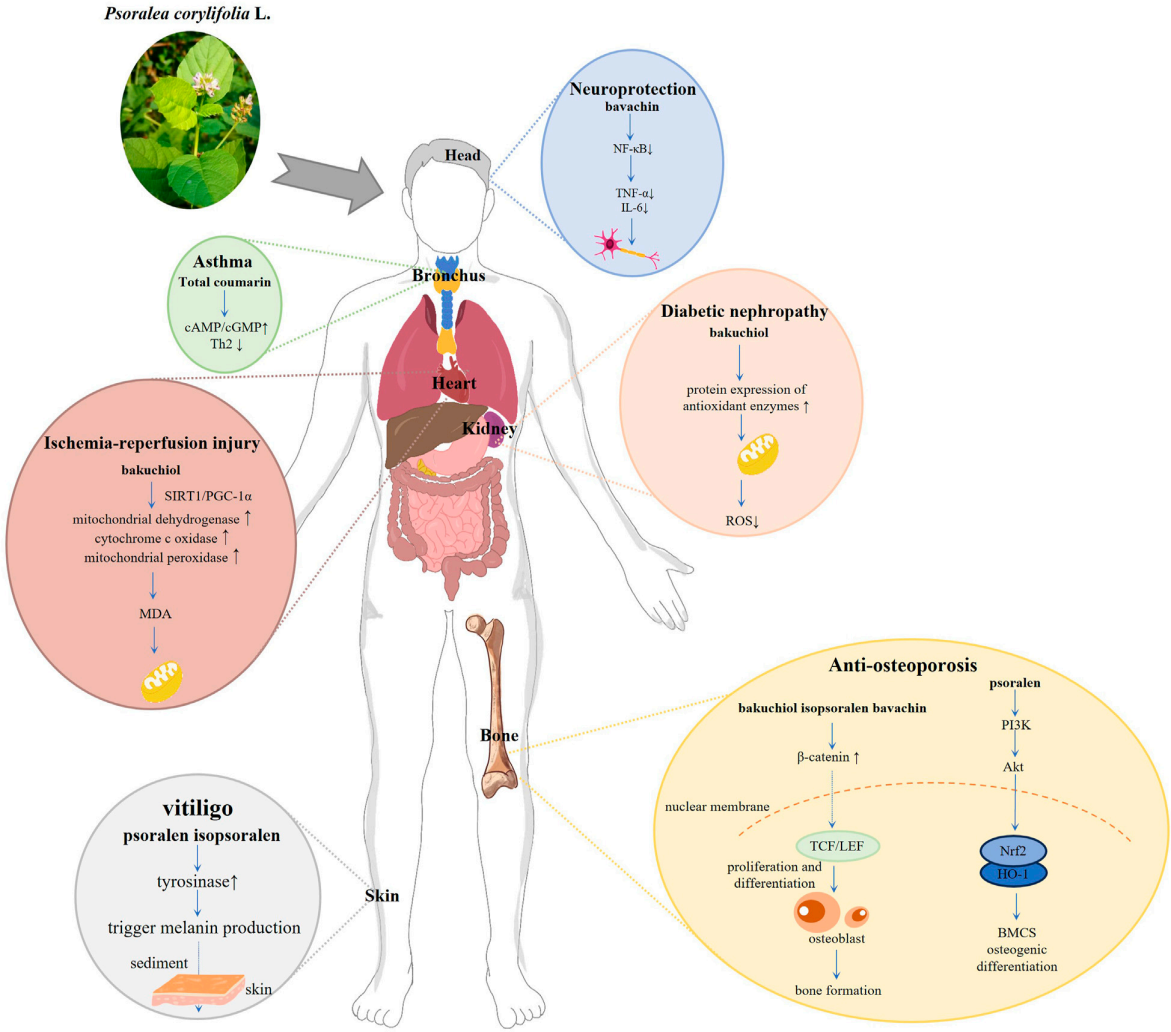
Pharmacological mechanism diagram of *Psoralea corylifolia* L.

The abundant and diverse active metabolites of PCL constitute the material basis for its pharmacological activity. PCL is frequently utilized in TCM to treat osteoporosis, and its effectiveness is notable. With the deepening of modern medical research, it has been widely explored and applied in the adjuvant treatment of tumor diseases, slowing down the aging process, has antibacterial, anti-inflammatory, and estrogen-like effects. Supplementary Table 2 provides a detailed overview of the pharmacological activity, modeling methods, and active metabolites of PCL, while Figure 3 illustrates the underlying mechanisms involved.

### 7.1 Anti-osteoporosis effects

Osteoporosis (OP) is a systemic bone disease characterized by reduced bone mass, density, microstructure, and fragility. Owing to the lack of obvious clinical symptoms in the early stages, it can quickly progress unnoticed. It is sometimes called a “silent disease” (Du and Li, 2022). While traditional anti-OP medications play a role in prevention and treatment, they often have various side effects, leading healthcare professionals to explore alternative plant-based therapies as a complement to Western medicine. According to TCM theory, the kidney is believed to support bone growth and development while maintaining bone health (Li et al., 2017c). PCL is a traditional remedy known for its ability to strengthen the kidney. The active metabolites found in PCL, including coumarin, flavonoids, and meroterpenes, are believed to fight OP by regulating the expression of essential proteins involved in different OP signaling pathways.

PCL primarily regulates the proliferation and differentiation of osteoblasts and osteoclasts. It increases bone formation and reduces bone absorption by modulating the expression levels of the Wnt/β-catenin, PI3K/Akt, PPAR-γ/Wnt, NF-κB and RANKL/RANK/MAPK signaling pathways, thus participating in the process of OP (Wu et al., 2023). Isopsoralen can effectively suppress oxidative stress and caspase-3/9 activity in postmenopausal OP mice, leading to an increase in the protein levels of Wnt/β-catenin and a decrease in the protein levels of PPAR-γ (Wang J. et al., 2018). Psoralidin effectively blocked the phosphorylation of NF-κB/p65 and the degradation of I-κB triggered by RANKL. Furthermore, it suppressed the expression of osteoclast marker genes such as TRAP, cathepsin K, and OSCAR (Kong et al., 2017). Although this study examined only the regulatory role of osteoclasts, it did not fully explore the potential of psoralidin in stimulating osteoblast differentiation.

With increasing age, oxidative stress can result in a decrease in osteoclast quantity and functionality (Lee et al., 2013). The PI3K/Akt signaling pathway is vital in cellular response to oxidative stress. When this signaling pathway is activated, it can inhibit the activity of GSK-3β and promote the activation of Nrf-2. Subsequently, the Nrf-2/HO-1 signalling pathway is activated, which increases the levels of SOD and GSH-Px and decreases the levels of ROS and MDA, thereby reducing the damage caused by oxidative stress (Ge X.-H. et al., 2018). Psoralidin can stimulate the downstream factor GSK-3β of the PI3K/Akt axis and enhance the Nrf-2/HO-1 pathway to accelerate the elimination of ROS (Zhai et al., 2018). Interacting with the estrogen receptor signaling pathway, increases Nrf2 expression, reduces ROS production, and effectively regulates oxidative stress levels, ultimately preventing bone loss (Zhai et al., 2017). Despite its promising effectiveness in combating osteoporosis, the dose-dependent properties of psoralidin have not been fully explored. As a result, it remains unclear how the biological activity of psoralidin may be influenced by varying doses, as well as whether an optimal range for dosage exists.

PCL has been found to have significant anti-OP effects in clinical trials. Studies have revealed that Xanling Gubao capsules, which contain psoralen as the primary metabolite, can reverse the methylation level of the Leptin promoter induced by ovarian extraction (Wang J. et al., 2021). This reversal promotes transcription and upregulates the expression of leptin, leading to an increase in the expression of core binding factor α1, alkaline phosphatase, osteocalcin, osteopontin, and type I collagen. These findings suggest a potential role for PCL in the prevention and treatment of postmenopausal OP. Moreover, certain clinical studies have indicated that PCL can significantly increase bone mineral density, decrease fracture risk, and enhance patients’ overall quality of life (Ye et al., 2023). Zhang et al. studied the impact of the metabolite Buguzhi granule on rats with osteoporosis caused by retinoic acid (Zhang et al., 2011). The study results revealed a decrease in the atrophy of gonadal organs, a decrease in serum alkaline phosphatase activity, and an increase in the ash weight and bone calcium content of rats with OP.

In summary, PCL has led to significant advancements in the treatment of osteoporosis. Current research primarily has focused primarily on individual metabolites of PCL, such as psoralen, isopsoralen, and psoralidin, with limited exploration of other metabolites, creating a research gap. Given the complex and diverse nature of PCL, intricate interactions between its various metabolites may warrant further investigation to understand its pharmacological effects.

### 7.2 Regulation of estrogen levels

Estrogen is crucial in controlling female reproductive system growth, development, and differentiation. When estrogen levels decrease, menopausal women may experience various climacteric syndromes, such as cardiovascular diseases, osteoporosis, and senile dementia (Li and Song, 2019). Exogenous estrogen acts as a double-edged sword, providing therapeutic benefits while also causing numerous adverse reactions. As a result, phytoestrogens that have minimal side effects are promising new approaches for treating illnesses (Li and Wei, 2020). Some important phytoestrogens, such as psoralen, isopsoralen, bavachin, neobavaisoflavone, corylifol A, and isobavachalcone, have been the focus of research (Xin et al., 2010). These metabolites can mimic the effects of estrogen in the body by binding to estrogen receptors and activating related signaling pathways.

The estrogenic impact of PCL helps stimulate osteoblast growth and differentiation while simultaneously inhibiting osteoclast function, leading to enhanced bone formation and reduced bone resorption (Li and Song, 2019). Isopsoralen, a coumarin estrogen, mimics estrogen’s effects by binding to estrogen receptors (Wang T. et al., 2020). Studies have shown that in models of osteoporosis caused by hormone deficiency, isopsoralen acts as an AhR antagonist, increases the expression of ERα, enhances bone strength and trabecular bone structure, and promotes osteoblast differentiation through the AhR/ERα pathway (Ge L. et al., 2018). While studies have shown that isopsoralen can interact with AhR and influence its function at the cellular level, the specific binding site has yet to be determined. ER may increase the expression of critical molecules such as β-catenin in osteoblasts by regulating the Wnt/β-catenin signaling pathway, thus promoting the proliferation and differentiation of osteoblasts. The increase of osteoblasts contributes to bone formation and increases bone mass (Liedert et al., 2020). The isoflavones found in PCL also display strong estrogen-like properties, contributing to their antiosteoporosis activity by activating the ER-Wnt-β-catenin signaling pathway (Cai et al., 2021). Lim et al. found that bakuchiols act like estrogen, binding firmly to ERα and effectively reducing postmenopausal bone loss (Lim et al., 2009). It is achieved by increasing alkaline phosphatase, calcium, serum E2, and bone mineral density levels while decreasing inorganic phosphorus levels.

Phytoestrogens can compete with animal estrogens for binding to estrogen receptors on target organs, potentially inhibiting the effects of animal estrogens and promoting the proliferation of tumor cells (Li and Wei, 2020). Li et al. found that a high concentration of bakuchiol can trigger the upregulation of ERβ and the downregulation of ERα in MCF-7 cells (Li et al., 2016). It led to the arrest of both MCF-7 and MDA-MB-231 cells in the S phase, ultimately causing apoptosis in MCF-7 cells and disrupting the mitochondrial membrane potential pathway, demonstrating its anti-breast cancer properties. Additional research suggests that the inhibitory effect of psoralen may also be linked to Erβ. Psoralen has a notable inhibitory effect on prostate cancer LNCa P-AD cells *in vitro* in a dose-time dependent manner (Chen S. et al., 2017). This founding offers a novel target and approach for prostate cancer treatment. The therapeutic role of PCL in endometrial cancer involves activating the estrogen receptor and regulating estrogen levels in the body (Jiang et al., 2024). However, there might be some variation from the *in vitro* experimental findings during the absorption and metabolism of these effective metabolites. Therefore, to accurately evaluate the biological activity and efficacy of PCL extracts, it is advisable to conduct comprehensive research combined with *in vivo* experiments.

5, 10 mg/kg isopsoralen was injected intraperitoneally every day for 2 weeks, which significantly enhanced the motor function of hind limbs in mice with spinal cord injury, and exerted estrogen-like neuroprotection by activating ERα and regulating PI3K/AKT pathway (Li et al., 2017b). Wu’s research focused on the effects of PCL extract on enhancing learning and memory in APP/PS1 transgenic mice and its correlation with the ERβ/ERK signaling pathway (Wu F. et al., 2022). This study revealed a significant increase in the protein expression of ERα and ERβ and the p-ERK/ERK ratio in the hippocampus of mice treated with PCL extract. Nonetheless, further investigations are needed to ascertain whether PCL extract can hinder microglia-mediated neuroinflammation through estrogen receptors and enhance neuronal synaptic plasticity.

Despite the pharmacological activity of PCL as a potential estrogen agonist in various aspects, its underlying mechanism of action and identification of specific molecular targets are still in the early stages of exploration. One of the key challenges in current research is determining the interaction between PCL and estrogen receptors, particularly with respect to the binding site and the comprehensive understanding of the dose-effect relationship.

### 7.3 Anti-tumor effects

Epithelial-mesenchymal transition (EMT) is an essential process of embryonic development and a critical process of cancer cell invasion and metastasis (Jung et al., 2015). Flavonoids derived from PCL exhibit remarkable antitumor activity by efficiently inhibiting epithelial-mesenchymal transition, thereby restraining the proliferation and metastasis of malignant tumors (Shi et al., 2024). Additionally, PCL can trigger apoptosis in tumor cells, suggesting a novel approach for treating tumors. Jung et al. revealed that the aqueous extract of PCL significantly reduced the expression of the EMT markers N-cadherin and vimentin caused by LPS while simultaneously increasing the expression of β-catenin via the NF-κB-SNAIL signaling pathway (Jung et al., 2015). This new evidence suggests that PCL impedes the invasion and migration of cancer cells by suppressing EMT. However, these studies investigated the influence of PCL extract on only the estrogen receptor without providing a definitive conclusion on which active metabolites are primarily responsible for this effect. Chen et al. discovered that corylin can trigger the growth inhibition-specific transcript 5 (GAS5) of antitumor lncRNAs, leading to the suppression of EMT (Chen C.-Y. et al., 2018). As a result, the proliferation, migration, and invasion of HCC cells are inhibited. Nevertheless, further investigation is needed to understand how corylin increases the expression of GAS5. Furthermore, the functions of numerous lncRNAs modulated by corylin in the antitumor mechanism remain unclear.

The release of proinflammatory cytokines by immune cells can promote tumor growth, but PCL has been shown to have an immunomodulatory effect. This effect can help restore abnormal cells and factors to their normal state, increase the body’s immune function and ultimately prevent tumor growth and metastasis (Li and Song, 2017). Research has shown that PCL extract can enhance the immune system of mice, suppress EAC peritoneal tumor growth, and increase various immune responses (Latha et al., 2000). Zhao et al. discovered that the presence of IL-6 in the colon tissue of rats exposed to bavachinin was suppressed, leading to increased p53 levels that triggered apoptosis (Zhao et al., 2021). PCL has various effects on immune organs, cells, and molecules, promoting or inhibiting them. These results offer a fresh perspective for discovering effective strategies for treating tumors.

Currently, research on the antitumor activity of PCL has focused on gastric, breast, prostate, liver, and rectal cancer, and researchers have use mostly *in vitro* cell models for verification. However, findings from *in vitro* studies may not wholly reflect the actual therapeutic effects and mechanisms of PCL in living organisms. Therefore, further research using animal models and clinical trials is necessary to better understand the antitumor effects of PCL. Furthermore, due to the low content of anti-cancer active metabolites in PCL and its simple structure, there is potential for enhancing its anticancer activity through structural modification and clinical verification. This approach offers a novel and efficient approach to tumor treatment.

### 7.4 Antibacterial and antifungal effects

The overuse of antibiotics has led to a severe global health threat of bacterial resistance to antibiotics. Owing to its rich plant resources, especially Chinese herbal medicines, China provides a unique opportunity for research on botanical bactericides. PCL has been shown to effectively inhibit and kill a variety of bacteria.

Psoralidin, bakuchicin, psoralin, and angericin in PCL exhibit solid antibacterial properties against gram-negative and gram-positive bacteria (Khatune et al., 2004). Yin et al. found that 16 neopentyl flavonoids isolated from PCL were tested for their antibacterial effects (Yin et al., 2004). Metabolites 5, 10, and 16 showed significant antibacterial activity against common pathogens, outperforming natural bacteriostats like lignan and magnolol. Nevertheless, these findings are derived from *in vitro* studies and have not been validated through *in vivo* experiments. Consequently, the true bacteriostatic impact and safety of PCL in clinical use require additional investigation and confirmation. The antibacterial mechanism of PCL is believed to involve damage to the bacterial cell membrane. Li et al. discovered that the ethanol extract of PCL can penetrate cell membranes and disrupt cellular metabolism by inhibiting ATP or interfering with the proton gradient (Li H.-N. et al., 2019). This leads to energy depletion and prevents bacteria from being expelled, ultimately causing irreversible damage to the plasma membrane and exhibiting antibacterial properties. The 90% ethanol extract of PCL exhibited a notable antibacterial effect against *M. abscessus*. Isobavachalcone was found to be particularly effective in disrupting the cell membrane structure of bacteria, leading to surface shrinkage and depression and a significant reduction in bacterial volume (Cao et al., 2024). However, the biological activity of PCL extract in the human body can be affected by numerous biological factors, causing its actual impact *in vivo* to differ from that observed *in vitro*, thus requiring validation *in vivo*. PCL has also shown efficacy against skin fungi. Research has shown that the antifungal properties of PCL are associated with the presence of flavonoids (Rajendra Prasad et al., 2004). In a guinea pig model of tinea pedis, bakuchiol was found to alleviate this condition by increasing the permeability of fungal membranes and promoting ROS production, ultimately reducing the fungal load on infected feet (Lau et al., 2014). However, to establish the practicality of external skin treatment, further studies are needed to assess the biological safety and transdermal capabilities of bakuchiol *in vitro*.

While PCL has shown potential for antibacterial activity, its specific mechanism of action and effectiveness still require further investigation and confirmation. Furthermore, we must consider the interaction between PCL and other medications, as well as the risk of drug resistance. Simply put, PCL holds great potential in the field of antibacterial research, with opportunities for enhancement through the exploration of combinations with other antibacterial drugs, the development of nanodrug delivery systems, and the optimization of its structure for increased practical application.

### 7.5 Antioxidant effects

The remarkable antioxidant activity of PCL has made it a potential treatment for numerous diseases. Its therapeutic effects have been particularly notable in conditions such as neurodegenerative diseases, cardiovascular diseases, diabetes and its complications, and skin aging. PCL extract has been shown to protect neuronal cells from damage caused by oxidative stress and lipotoxicity (Lee et al., 2016). It significantly increases the viability of PC12 cells treated with PA, has antiapoptotic effects, reduces the production of reactive oxygen species induced by PA, and increases the expression of antioxidant genes. However, the exact cause of the neuroprotective benefits of the extract is still unknown-whether it is due to a single metabolite or the combined effects of several metabolites.

Oxidative stress is a crucial factor in the development of cardiovascular diseases (Wang and Kang, 2020), and the antioxidant properties of PCL can help reduce oxidative damage to the cardiovascular system, thereby preventing conditions such as atherosclerosis and myocardial ischemia. Bakuchiol has been found to have a protective effect on the heart. Research by Feng has shown that bakuchiol increases the activity of certain enzymes in the mitochondria, which helps reduce oxidative damage and alleviates ischemia-reperfusion injury (Feng et al., 2016). Additionally, bakuchiol activates the SIRT1/PGC-1α signaling, leading to a decrease in malondialdehyde production. However, the study only employed a single dose without systematically examining dose-dependent effects.

PCL also has potential therapeutic benefits for diabetes and its complications. By reducing oxidative stress in pancreatic islet cells and surrounding tissues, bakuchiol may improve insulin sensitivity and lower blood sugar levels. In diabetic mice induced by streptozotocin, oral administration of bakuchiol extract significantly improved hyperglycemia (Seo et al., 2014). PCL extraction improved glucose tolerance, the serum insulin level, and the preventive effect of psoralen and isopsoralen H_2_O_2_ on β-cell death. These findings suggest that PCL extract may be a potential pharmacological drug that can prevent pancreatic β-cell damage caused by oxidative stress related to diabetes. Diabetic nephropathy (DN) is a significant complication of diabetes. Bakuchiol upregulates the protein expression of antioxidant enzymes in kidney tissue, reduces the production of mitochondrial ROS, and improves DN by inhibiting oxidative stress and enhancing mitochondrial function (Park et al., 2023).

Additionally, PCL may help slow skin aging and maintain firm and radiant skin. Keap1-Nrf2 signaling pathway is an important intracellular antioxidant stress pathway which can maintain skin homeostasis and play an essential role in many skin diseases (Baird and Yamamoto, 2020). Corylin, a potent antioxidant, can inhibit oxidative stress in mouse cells through the Keap1-Nrf2 pathway, thus preventing skin aging and preserving cell vitality (Li N. et al., 2022). Despite exhibiting significant antioxidant activity *in vitro*, examining the transdermal absorption rate, absorption capacity, and distribution of corylin post-absorption is crucial to ensure its extended use safety. Bakuchiol, known for its potent antioxidant properties, also shows promise as a natural food preservative, surpassing traditional preservatives in preventing enzyme-catalyzed food browning (Cariola et al., 2023). Its potential as a natural food preservative could open new possibilities for its use in the food industry.

While PCL has excellent potential for antioxidation, more in-depth research on its antioxidation mechanisms is needed. While the scavenging ability of ABTS free radicals and DPPH free radicals can provide some insight into the antioxidant capacity of PCL, more studies are needed to determine its antioxidant potential fully. Future studies should explore the specific molecular mechanisms of the antioxidation properties of PCL and its therapeutic effects in different disease models. Furthermore, the bioavailability and safety of PCL require further investigation to support its clinical application. As research progresses, the application possibilities of PCL in the field of antioxidants will continue to expand.

### 7.6 Anti-inflammatory effects

The anti-inflammatory effect is a significant pharmacological benefit of PCL. MAPK pathway is the most critical regulator of proinflammatory cytokines, which plays an important role in regulating neuroplasticity and maintaining the inflammatory response. Inhibition of the ERK, p38, and JNK MAPK pathways can alleviate inflammation and neuropathic pain (Ji et al., 2009). Bakuchiol, a metabolite of PCL, has been shown to effectively reduce the production of PGE2, TNF-α, and IL-6 in BV-2 cells stimulated with LPS through the p38 MAPK and ERK pathways without causing any cytotoxic effects (Lim et al., 2019). Nevertheless, the study did not clearly determine the optimal dosage of bakuchiol for its anti-inflammatory effect. Studies have also highlighted the potent anti-inflammatory activity of chalcone metabolites with hydroxyl, methoxy, carboxyl, isoprene, and heterocyclic substituents (Mahapatra et al., 2017). Isodorsmanin A, a chalcone metabolite found in PCL, has been investigated for its anti-inflammatory properties, particularly its ability to inhibit inflammatory mediators and proinflammatory factors by targeting the JNK and NF-κB signaling pathways in LPS-stimulated macrophages (Chung et al., 2023). However, the study did not extensively explore the structure-activity relationship between the chemical structure of isodorsmanin A and its anti-inflammatory activity, underscoring the necessity for future investigations to uncover the intrinsic link between its chemical composition and anti-inflammatory effects.

PCL has demonstrated anti-inflammatory and pain-relieving properties in treating rheumatoid arthritis, neuritis, periodontitis, and various other diseases. Huang et al. utilized LPS to stimulate BV2 cells, mimicking the inflammatory conditions in the brain (Huang et al., 2018). He found that corylin exhibited anti-inflammatory properties by suppressing the activation of NLRP3 inflammatory bodies and reducing LPS-induced inflammation. This suggests that corylin is a potential treatment for brain inflammation and could slow the progression of neurodegenerative diseases. Liu et al. conducted a study on how psoralen affects the intestinal barrier and alveolar bone loss in rats with chronic periodontitis (Liu et al., 2021). These findings indicate that psoralen can affect both the intestinal immune barrier and the ecological barrier. It can also regulate the immune response by increasing the secretion of the anti-inflammatory factor IL-10 while decreasing the secretion of the proinflammatory factor TNF-α. This ultimately leads to a reduction in alveolar bone loss in rats with experimental periodontitis. Pro-inflammatory cytokines can activate the JAK/STAT signaling pathway. Once the JAK/STAT signaling pathway is out of balance, the inflammatory response will accelerate (Malemud and Pearlman, 2009). Wang et al. also verified that psoralen B can suppress the proliferation, migration, and invasion of MH7A cells triggered by TNF-α (Wang et al., 2022). It can induce apoptosis, decrease inflammation, and function as an antirheumatoid arthritis agent by regulating the PI3K/AKT and JAK/STAT signaling pathways.

Despite strong evidence from experimental studies showing the significant anti-inflammatory effects of PCL and its extracts in various inflammatory models, more systematic clinical trials are necessary to validate their safety and effectiveness. Additionally, further exploration is needed to understand its specific mechanism of action and identify the most suitable medication regimen for different diseases.

### 7.7 Neuroprotective effects

As the population continues to age, the occurrence of neurodegenerative diseases is increasing, presenting a significant challenge to the elderly population. Research has shown that PCL can protect against conditions such as Alzheimer’s disease, Parkinson’s disease, and stroke. Its potential mechanisms may be linked to a reduction in inflammatory reactions, inhibition of oxidative stress, enhancement of mitochondrial dysfunction, and improvement in the survival and function of nerve cells.

Chen et al. discovered that total isopentenyl flavonoids extracted from PCL significantly increased various AD markers in the brains of SAMP8 mice, reduced the expression of proinflammatory cytokines, and lowered the level of oxidative stress biomarkers *in vivo* (Chen Z.-J. et al., 2018). This leads to improved cognitive ability and a potential role in preventing AD. Psoralen can regulate both the Nrf2/HO-1 and NF-κB signaling pathways. It can inhibit the production of ROS and NO while also significantly increasing the activities of CAT and SOD. Additionally, psoralen promotes GSH secretion and inhibits the secretion of inflammatory factors, ultimately improving LPS-induced neuroinflammation (Wang et al., 2023d). The ethanol extract of PCL effectively diminishes the inhibition of 3-NP-induced mitochondrial dysfunction in PC12 cells. It decreases the mitochondrial superoxide level while also uncoupling and stimulating mitochondrial respiration. Additionally, it increases the biological energy storage capacity, making it a promising drug for treating neurodegenerative diseases (Im et al., 2014). While this study focused primarily on the verification stage of cell models, investigations into the signaling pathways and regulatory genes upstream of cells still need to be completed.

The activation of NF-κB may contribute to the secretion of pro-inflammatory cytokines. MAPK is considered a classic way to start NF-κB (Li M. et al., 2022). Psoralen inhibits the activation of the MAPKs and NF-κB signaling pathways by blocking the phosphorylation of Fyn and PKCδ in microglia. This inhibition leads to reduced expression of proinflammatory cytokines, ultimately helping to decrease neuroinflammation and alleviate oxidative stress (Guo et al., 2024).

Research has shown that PCL positively affects nerve development, learning, and memory. These effects are believed to be mediated through improvements in mitochondrial function, reductions in peripheral histamine levels, the regulation of energy metabolism, and antioxidant levels. In studies conducted on APP/PS1 mice, PCL was found to significantly improve dark avoidance latency and reduce the number of mistakes made (Tong et al., 2020). Additionally, PCL was shown to enhance new object recognition. Metabolic pathway analysis revealed that PCL primarily impacts histidine metabolism, the citric acid cycle, taurine and hypotaurine metabolism, and glucose metabolism to increase learning and memory (Qiao et al., 2023). While this study has extensively focused on metabolism, it has yet to discover and specify relevant biomarkers.

In conclusion, while PCL shows promise in neuroprotection research, further studies are necessary to fully comprehend its mechanism of action and potential applications. Furthermore, it is vital to research how the active metabolites of PCL are distributed and metabolized in the body and to formulate these active metabolites precisely into liposomes, nanoparticles, and other forms to facilitate their passage through the blood-brain barrier and improve their effectiveness.

### 7.8 Other effects

PCL has been extensively utilized in the treatment of vitiligo, asthma and vascular diseases. In PCL, psoralen and isopsoralen are metabolites sensitive to light and absorb ultraviolet rays. They promote melanin growth in the skin and store it beneath the skin, ultimately helping to restore the natural color of the skin in vitiligo-affected areas (Huang F. et al., 2021). This treatment is commonly used in clinical settings.

Asthma is fundamentally a persistent allergic-inflammatory condition affecting the airways (Lin, 2000). Tonifying the kidney for asthma treatment has always been a top recommendation from the TCM family. PCL is commonly prescribed for asthma treatment. The administration of total coumarin from PCL significantly increased the serum cAMP/cGMP ratio in asthmatic rats, providing therapeutic benefits for bronchial asthma (Yu et al., 2006). Psoralen can suppress hyperresponsiveness and airway inflammation in a rat asthma model by inhibiting the Th2 response (Jin et al., 2014).

Kassahun Gebremeskel et al. found that specific metabolites in PCL, including bakuchiol, isobavachalcone, isopsoralen, and psoralen, can cause arterial vasodilation in rats by affecting both endothelium-dependent and independent mechanisms (Kassahun Gebremeskel et al., 2017). These metabolites also inhibit TRPC3 channel activity and prostaglandin secretion and alleviate pH-induced vasoconstriction. Bakuchin, in particular, was found to induce vasodilation by activating the endothelium-dependent NO-cGMP signal transduction pathway (Li et al., 2011). This discovery suggests that PCL could be a promising botanical drug treatment for vascular conditions such as atherosclerosis and hypertension and may have potential in treating vascular diseases. Some metabolites in PCL have also been found to inhibit tumor angiogenesis, which could disrupt the nutrient supply to tumors and hinder their growth. After treatment with PCL, there was a significant decrease in Ki-67, CD31, and VEGF levels in tumor tissues and a reduction in serum IL-4 levels in tumor-bearing mice (Amani et al., 2021).

## 8 Hepatotoxicity

Owing to its unique metabolites, PCL has shown remarkable bioactivity, providing new ideas for the treatment and prevention of a variety of diseases. However, as the scope of the clinical application of PCL continues to broaden and the dosage and frequency of use increase, its potential toxic effects have gradually been revealed. The National Adverse Drug Reaction Monitoring Center has issued multiple warnings about the potential for liver injury from the use of PCL and its related products, such as Zhuanggujie Pill, Xianlinggubao Capsule, and Zhikang Capsule (Feng et al., 2023). This has created significant challenges for the safe clinical use of PCL. To gain a deeper understanding of PCL hepatotoxicity, keyword co-occurrence analysis was conducted on Chinese and English documents via VOS viewer software. Supplementary Figures 3A, B show the keyword co-occurrence maps of Chinese and English keywords in the research field of PCL hepatotoxicity, respectively.

### 8.1 Hepatotoxic metabolites

Currently, hepatotoxic elements in PCL, which result in the main adverse effects of liver injury, may predominantly include psoralen, isopsoralen, bakuchiol, bavachin, bavachinin, and other related metabolites.

Sun et al. discovered that hepatotoxic metabolites are associated primarily with three pathways: the p38/JNK-MAPK pathway, which is linked to oxidative stress in the endoplasmic reticulum; the PI3K-AKT-mTOR pathway, which is connected to mitochondrial damage; and the NLRP3 pathway, which is associated with immune stress (Sun et al., 2023). However, even though this study has provided a prediction of the hepatotoxic action pathway and action site of PCL, these findings have yet to be confirmed through experimental testing. The water extract contains psoralenoside, isopsoralenoside, psoralen, and isopsoralen, which are all potentially hepatotoxic (Zhou et al., 2013). Following a 12-week experiment involving rats orally administered a water extract of PCL, liver enlargement, an increased liver coefficient, and moderate diffuse steatosis were observed. Nevertheless, the connection between hepatotoxicity, dosage, and duration of PCL has yet to be fully elucidated. Bavachinin has been shown to induce apoptosis and necrosis in hepatocytes by damaging mitochondria (Ji et al., 2018). Wang et al. discovered that various metabolites of PCL, including psoralen, isopsoralen, corylifolin, bakuchiol, and psoralidine, exhibited strong inhibitory effects on L02 liver cells (Wang X. et al., 2020). These metabolites were identified as potential factors contributing to liver cell damage induced by PCL, with corylifolin and psoralen showing stronger hepatotoxicity than other metabolites.

However, current research is limited to *in vitro* toxicity assessments of PCL, and corresponding animal experimental and clinical data are needed. Additionally, more research is needed to determine whether any other chemical metabolites may pose a hepatotoxic risk.

### 8.2 Hepatotoxic mechanism

Liver injury is a key factor in liver disease, and its mechanism is complex and plays a role throughout the progression of liver disease. The toxicological mechanism of psoralen and its preparations may involve disruptions in bile acid transport, oxidative stress in the endoplasmic reticulum, mitochondrial damage, and alterations in energy metabolism pathways (Duan et al., 2020).

#### 8.2.1 Bile acid transport-efflux balance

Cholestasis is characterized by disruption of normal bile secretion and excretion, resulting in bile accumulation in the bloodstream instead of flowing into the duodenum (Hilscher et al., 2020). Excess bile acids can lead to immune system dysfunction, inflammation, and fibrosis, ultimately causing damage to liver cells and the entire body (Chen and Zhang, 2023; Dong et al., 2023).

Psoralen reduced the transcription and expression of the bile salt export pump and increased the transcription and expression of Na^+^-sodium taurocholate cotransport after 24 h, which led to increased bile acid transport into hepatocytes, and ultimately led to cholestatic liver injury (Wang et al., 2015). Further research revealed that PCL can inhibit the expression of FXR and PPARα, causing bile acid and lipid metabolism disorders (Gao et al., 2024). This further activates the TNF-α/NF-κB inflammatory pathway, damaging hepatocytes and causing liver inflammation and injury. Research findings indicate that the protein levels of BSEP and NTCP in mouse livers significantly decrease after 4 weeks of continuous administration of an aqueous PCL extract (Bi et al., 2015). These finding suggest that the extract inhibits the absorption and excretion of bile acids in hepatocytes, leading to cholestasis and hepatocyte damage due to increased bile acid content. Xu et al. found a “dose-hepatotoxicity” relationship after the administration of an aqueous extract of PCL. The expression of the BSEP and FXR genes and proteins in rats decreased significantly. In contrast, the expressions of the CYP7A1, TNF-α, and NF-κB genes and proteins increased significantly, which affected the secretion and excretion of bile acids in the liver and induced inflammation (Xu B. et al., 2023). Notably, the ethanol extract of PCL can lead to cholestatic liver injury related to sex differences, especially in female rats (Wang et al., 2012). This discovery provides a new perspective and way of considering the safety of PCL in clinical applications.

The studies mentioned above focused only on the hepatotoxicity properties of PCL extracts. However, this extract contains multiple metabolites that interact in complex ways, potentially intensifying its hepatotoxic effects. The exact mechanism behind this interaction and how it enhances hepatotoxicity remain unclear.

#### 8.2.2 Oxidative stress pathway

Oxidative stress is a significant factor in the development of different liver diseases. While normal liver cells produce a small amount of ROS (Ramachandran and Jaeschke, 2018), excessive inhibition of GSH can result in increased ROS production, leading to liver cell damage and death (Tang et al., 2024).

The activation of the oxidative stress response pathway by PCL and its monomeric metabolites results in the generation of a significant amount of ROS, depleting GSH and causing mitochondrial dysfunction, ultimately resulting in liver injury (Feng et al., 2023). By activating the p38/JNK-MAPK signaling pathway, bavachinin induces excessive ROS in HepaRG cells, which results in oxidative stress damage (Wang S. et al., 2018). Psoralen binds to CYP2D6, CYP3A4, GST-α, and GST-μ, which leads to the inhibition of their activities. Consequently, this inhibition causes GSH depletion *in vivo*, ultimately resulting in liver damage (Jiang et al., 2022). Additionally, the hepatotoxic effects of PCL can be attributed to the presence of 4-hydroxylonchocarpin and corylifolia A. These metabolites increase MDA and ROS levels, decrease SOD activity and GSH levels, and reduce the mitochondrial membrane potential. These alterations ultimately result in oxidative stress and mitochondrial dysfunction, leading to liver cell injury and apoptosis (Ouyang et al., 2023). Bavachin, psoralidin, neobavaisoflavone, and bakuchiol have all been identified as metabolites that can increase intracellular lipid accumulation and ROS levels, ultimately causing greater harm to the liver (Guo et al., 2021).

Nevertheless, the findings from the aforementioned study have been confirmed only at the cellular level, and further validation through more extensive animal studies is still needed. Moreover, the current assessment methods predominantly depend on alterations in MDA, SOD, GSH, and other markers, indicating the need for a more thorough investigation into the underlying mechanism involved.

#### 8.2.3 Mitochondrial damage and the endoplasmic reticulum stress pathway

The overabundance of ROS results in endoplasmic reticulum stress, causing imbalances in Ca^2+^ levels and activating IRE1α, PERK, and ATF6. This leads to cell death and disruptions in the normal functioning of liver cells (Villanueva-Paz et al., 2021). *In vivo* experiments revealed that the administration of 80 mg kg^−1^ psoralen to mice for 3 days led to a significant increase in the levels of markers associated with endoplasmic reticulum stress, including IRE1α, PERK, and ATF6 (Yu et al., 2022). Isobavachalcone causes mitochondrial damage and liver toxicity through the accumulation of ROS and the inhibiting of Akt, leading to cell death and apoptosis in HepG2 cells (Zhao et al., 2022). Psoralen A at a concentration of 20 μ mol L^−1^ leads to the accumulation of ROS, a reduction in MFN2, and the activation of phosphorylated Akt. Consequently, this leads to endoplasmic reticulum stress, which ultimately inhibits cell proliferation (Yang et al., 2018). HCS technology and molecular docking analysis confirmed the high binding affinity of bavachin, isobavachalcone, bakuchiol, psoralidin, and other metabolites with mitochondrial respiratory complexes, indicating their role as potent mitochondrial hepatotoxic metabolites (Shang et al., 2024).

#### 8.2.4 Energy metabolism pathway

PCL is famous for its dryness and heat, which could impact energy metabolism pathways like amino acid and phospholipid metabolism in the liver. Zhang et al. identified 17 endogenous metabolic markers in rats with kidney yin/yang deficiency syndrome and reported that PCL regulates pathways such as amino acid and phospholipid metabolism (Zhang et al., 2023). Furthermore, this study suggested that kidney-yin deficiency syndrome and kidney-yang deficiency syndrome could be potential syndromes linked to liver damage and liver protection from PCL. This aligns with historical warnings in ancient medical texts about the risks of taking PCL for individuals with yin deficiency and excessive fire (Wu W. et al., 2022). The alcohol extract of PCL was found to cause specific liver injury by increasing the expression of certain proteins in rat liver tissue, indicating a potential connection between immune stress and energy metabolism. This mechanism may be closely related to the interaction between immune stress and energy metabolism (Chen X. et al., 2023). Hu et al. used the metabonomics method to preliminarily identify five biomarkers: indole, L-phenylalanine, sphingosine, lactic acid, and germine A (Hu et al., 2016). These markers offer new insights for future research on the effectiveness and hepatotoxic mechanisms of PCL.

### 8.3 Attenuation

By combining with various TCMs, it can significantly decrease the liver damage caused by PCL, which is crucial for ensuring the safety of its clinical application (Guo et al., 2017).

The combination of *Scutellaria baicalensis* Georgi, *Paeonia lactiflora* Pall. and PCL can reduce the exposure of psoralen and isopsoralen, the main hepatotoxic metabolites in PCL, and keep them at relatively stable levels *in vivo* (Zhai et al., 2023). When PCL is combined with *Polygonum multiflorum* Thunb., Rehmanniae Radix Preparata and Schisandra chinensis Fructus, it can help improve liver health by activating the AMPK/GSK-3β/Nrf2 signaling pathway and reducing oxidative stress (Wang et al., 2023b). The combination of Schisandrae chinensis and PCL can also help protect liver cells from oxidative damage and endoplasmic reticulum stress. Additionally, a study by Cai showed that combining walnut kernels, nutmeg, and PCL at a specific ratio can significantly reduce the hepatotoxicity of PCL, especially when walnut kernels are included (Cai, 2021). These findings provide valuable insights for the safe and effective use of PCL in clinical practice.

Although PCL is usually safe to use, its hepatotoxicity can potentially increase when it is mixed with other Chinese medicines. The pairing of PCL with Epimedium in TCM has been shown to result in liver injury. Research by Huang discovered that the combination of Epimedium and PCL in the Xianling gubao recipe can be more hepatotoxic than the entire recipe itself (Huang Y. et al., 2021). However, certain other metabolites in the formula, such as *Dipsacus asper* Wall. ex Henry, *Anemarrhena asphodeloides* Bge., *Rehmannia glutinosa* Libosch., and *Salvia miltiorrhiza* Bge., can help mitigate the liver damage caused by PCL and Epimedium. *Salvia miltiorrhiza* Bge., in particular, has shown the most effective protective effect. When PCL is used in conjunction with Epimedium, it can trigger specific liver toxicity under conditions of immune stress, leading to more severe harm in rats treated with both substances than in those treated with PCL alone (Gao et al., 2020).

Therefore, when PCL is used in combination with other botanical drugs, it is important to be mindful of the potential risk of increased toxicity to ensure the safety and efficacy of the medication.

## 9 Conclusion and prospects

PCL, a TCM commonly used as a tonic, has been utilized in clinical practice for centuries in China. Although significant progress has been made in PCL research, there are still numerous pressing issues that need to be addressed. This review provides an in-depth discussion of the current deficiencies in the study of PCL, and proposes its own views and solutions:

In the field of phytochemistry, there are still unidentified metabolites in PCL, but owing to their low content and difficult separation, they have not been thoroughly studied. In the future, multidimensional chromatographic separation can be used to improve the detection sensitivity and separation efficiency of trace components to construct a more complete chemical composition spectrum of PCL.

Currently, most pharmacological research has focused on the cellular and molecular levels, with limited research on the clinical application of PCL, hindering its development and widespread utilization. In addition to traditional applications, the potential therapeutic value of PCL in other diseases has been explored. Potentially effective disease models were screened through preclinical studies, and then, clinical observational studies and small-scale clinical trials were gradually carried out. In addition, studies on the combined application of PCL with other drugs can be carried out to assess drug interactions, enhancement of efficacy or changes in adverse reactions.

Although the hepatotoxicity of PCL has been reported, the cumulative toxicity resulting from the long-term use of PCL in small doses and the interactions when PCL is used in combination with other drugs have not been thoroughly studied. Moreover, the toxicity of PCL may also be affected by individual differences, dosage, duration of use and other factors. In addition, the mechanisms of toxicity of the various metabolites of PCL are complex and diverse, and there may be synergistic or antagonistic effects between these metabolites. In the future, investigating the relationship between the toxic effects of PCL and the mechanism of action and toxicity of PCL at the gene, protein, metabolite and other levels is necessary. In addition, pharmacokinetic and toxicokinetic studies should be carried out to provide more comprehensive data to support rational drug use and drug development.

In the present day, PCL has become an essential and vital medication in the annals of TCM, ushering in a new era in history. Put simply, as scientific and technological advancements continue and research becomes more profound, PCL will play an even larger role and make significant contributions to human health.
