# Supplementary material for: A review of Psoralea corylifolia L.: a valuable plant with profound biological significance
Source: Front Pharmacol. 2025 Jan 20;15:1521040. doi: 10.3389/fphar.2024.1521040 (PMC11788583; doi:10.3389/fphar.2024.1521040)
Supplement: Supplementary file 1 [file Table1.pdf]

Supplementary Table 1 Structures of metabolites isolated from *Psoralea corylifolia* L.

Category	No.	Metabolite	Formula	Molecular weight	Reference
Coumarins	1	psoralen	$C_{11}H_6O_3$	186.0317	(Khatune et al., 2004)
	2	isopsoralen	$C_{11}H_6O_3$	186.0317	
	3	8-methoxy psoralen	$C_{12}H_8O_4$	216.0423	(Ji and Xu, 1995)
	4	5-methoxy psoralen	$C_{12}H_8O_4$	216.0423	(Kulikov et al., 2022)
	5	bakuchicin	$C_{11}H_6O_3$	186.0317	(Khatune et al., 2004)
	6	psoralenoside	$C_{17}H_{18}O_9$	366.0951	(Qiao et al., 2006)
	7	isopsoralenoside	$C_{17}H_{18}O_9$	366.0951	
	8	psoralidin	$C_{20}H_{16}O_5$	336.0998	(Khatune et al., 2004)
	9	isopsoralidin	$C_{20}H_{16}O_5$	336.3	(Khastgir et al., 1961)
	10	corylidin	$C_{20}H_{16}O_7$	368.0896	(Gupta et al., 1977)
	11	psoralidin-2′,3′-oxide	$C_{24}H_{20}O_8$	436.4	(Gupta et al., 1980)
	12	3″,4″-dehydroisopsoralidin	$C_{20}H_{14}O_5$	334.33	(Yang et al., 2024)
	13	bvacoumestan A	$C_{20}H_{16}O_6$	352.0947	(Gupta et al., 1990)
	14	bvacoumestan B	$C_{20}H_{16}O_6$	352.0947	
	15	bavacoumestan C	$C_{20}H_{16}O_7$	368.3	(Chai, 2020)
	16	epi-bavacoumestan C	$C_{20}H_{16}O_7$	368.3	(Zhu et al., 2019)
	17	3″-methoxy-bavacoumestan C	$C_{21}H_{18}O_7$	382.3	(Lin et al., 2018)
	18	bavacoumestan D	$C_{20}H_{16}O_5$	336.3	(Chai, 2020)
	19	bavacoumestan E	$C_{21}H_{18}O_5$	350.37	(Zhu et al., 2019)
	20	neopsoralen	$C_{17}H_8O_5$	293.0372	(Peng et al., 1996)
	21	(E)-5-methoxy-8,8-dimethyl-3-[2-methyl-pent-3-en-2-yl-6-phenylpyrano[3,2-g] chromen-2(8H)-one	$C_{27}H_{28}O_4$	416.52	(Srinivasan and Sarada, 2012)
	22	7,2′,4′-trihydroxy-3-aryl-coumarin	$C_{15}H_{10}O_5$	270.0528	(Limper et al., 2013)
	23	psoracoumestan	$C_{20}H_{14}O_5$	334.3	
	24	pyranocoumarin	$C_{20}H_{18}O_4$	322.4	(Srinivasan and Sarada, 2012)
	25	4,5′,8-trioxsalen	$C_{14}H_{12}O_3$	228.2	(Zhang et al., 2017)
	26	sophoracoumestan A	$C_{20}H_{14}O_5$	334.0841	(Gupta et al., 1990)
	27	6-hydroxy-7-prenyl-coumarin	$C_{14}H_{14}O_3$	230.26	(Zhao, 2023)
	28	bavacoumestan F	$C_{20}H_{14}O_6$	350.33	(Xu et al., 2022)
	29	bavacoumestan G	$C_{22}H_{20}O_7$	396.40	
	30	bavacoumestan H	$C_{20}H_{16}O_7$	368.34	
	31	bavacoumestan I	$C_{20}H_{16}O_7$	368.34	
	32	bavacoumestan J	$C_{20}H_{16}O_6$	352.34	

	33	bavacoumestan K	$C_{20}H_{18}O_8$	386.36	(Yang et al., 2024)
	34	bavacoumestan L	$C_{20}H_{14}O_5$	334.33	
	35	coumesterol	$C_{15}H_8O_5$	268.22	
	36	3′-methoxycoumestrol	$C_{16}H_{10}O_6$	298.25	
	37	astragalin	$C_{21}H_{20}O_{11}$	446.0849	(Ji and Xu, 1995)
	38	3,5,3′,4′-tetrahydroxy-7-methoxyflavone-3′-o-α-l-xylopyranosyl(1→3)-o-α-larabinopyranosyl(1→4)-o-β-d-galactopyranoside	$C_{32}H_{38}O_{20}$	742.1956	(Yadava and Verma, 2005)
	39	corylifol C	$C_{21}H_{18}O_5$	338.1154	(Yin et al., 2004)
	40	coryfolia D	$C_{20}H_{16}O_5$	336.0998	(Yang et al., 2009)
	41	5,4′-dihydroxy-6,7-[(1′S,2′R)-1′-hydroxy-2′-(1-hydroxy-1-methylethyl)-furano]-flavonol	$C_{20}H_{18}O_8$	386.36	(Xu et al., 2021a)
	42	neoschaftoside	$C_{26}H_{28}O_{14}$	564.50	(He et al., 2021)
	43	4′-methoxyflavone	$C_{16}H_{12}O_3$	252.0786	(Rajendra Prasad et al., 2004)
	44	bavachin	$C_{19}H_{19}O_4$	324.1362	(Bhalla et al., 1968)
	45	bavachinin	$C_{20}H_{21}O_4$	338.1518	
	46	isobavachin	$C_{20}H_{19}O_4$	324.1362	
	47	6-prenylnaringenin	$C_{20}H_{20}O_5$	340.1311	(Matsuda et al., 2007)
Flavonoids	48	3′-hydroxyl isobavachin	$C_{20}H_{20}O_5$	340.38	(Yin et al., 2004)
	49	2(R,S)-4′-hydroxy-6,7-[2-(1-methoxy-1-methylethyl)-furano]-flavanone	$C_{21}H_{20}O_5$	352.1307	(Ma et al., 2016)
	50	brosimacutin E	$C_{20}H_{20}O_5$	340.38	(Takashima and Ohsaki, 2002)
	51	chromenoflavanone	$C_{20}H_{18}O_4$	322.1205	(Krishnamurti and Parthasarathi, 1981)
	52	bakuflavanone	$C_{20}H_{20}O_5$	340.10	(Cui et al., 2015)
	53	(2S)-7-methoxy-6-(2-hydroxy-3-methylbut-3-en-1-yl)-2-(4-hydroxyphenyl) chroman-4-one	$C_{21}H_{22}O_5$	354.40	(Zhu et al., 2019)
	54	corylifol F	$C_{17}H_{14}O_5$	298.2	(Du et al., 2019)
	55	corylifol H	$C_{20}H_{18}O_5$	338.36	(Liu et al., 2021)
	56	1″-methoxy-6,7-furanflavanone	$C_{21}H_{22}O_6$	370.40	(Zhu et al., 2019)
	57	2(*S*)-4′-hydroxy-7-methoxy-6- (1″, 2″-epoxy-3″-hydroxy dimethyl) flavanone	$C_{21}H_{22}O_6$	370.40	
	58	2(S)-4'-hydroxy-6-methoxy-	$C_{21}H_{20}O_5$	352.39	(Zhao, 2023)

	7-(2"-carbanyl-3"-methybult-3"enyl)-flavanone			
59	(2S)-7,4′-dihydroxyl-6-(2″,3″-epoxy-3″-methylbutyl)-flavanone	$C_{20}H_{20}O_5$	340.38	(Liu et al., 2018)
60	(2*S*) -4′-hydroxyl-7-hydroxymethylene-6-(2″, 3″-epoxy-3″-methylbutyl) -flavanone	$C_{21}H_{22}O_5$	354.40	(Zhu et al., 2019)
61	2(S)-6-methoxy-7-hydroxymethylene-4′-hydroxyl-flavanone	$C_{17}H_{16}O_5$	300.31	(Lin et al., 2018)
62	bavachinone A	$C_{20}H_{18}O_5$	338.36	(Li, 2019)
63	bavachinone B	$C_{20}H_{16}O_5$	336.34	
64	bavachinone C	$C_{20}H_{18}O_5$	338.36	
65	bavachinone D	$C_{17}H_{12}O_4$	280.28	
66	bavachinone E	$C_{20}H_{18}O_6$	354.36	
67	bavachinone F	$C_{20}H_{18}O_5$	338.36	(Xu et al., 2022)
68	bavachinone G	$C_{20}H_{18}O_5$	338.36	
69	bavachinone H	$C_{21}H_{22}O_5$	354.40	
70	bavachinone I	$C_{17}H_{16}O_6$	316.31	
71	bavachinone J	$C_{21}H_{24}O_7$	388.42	
72	furano-(2″,3″:7,6)-4′-hydroxyflavanone	$C_{17}H_{12}O_4$	280.28	
73	abyssinone I	$C_{20}H_{18}O_4$	322.36	
74	2*S*-abyssinone II	$C_{20}H_{20}O_4$	324.38	
75	3′-hydroxylbavachin	$C_{20}H_{20}O_5$	340.38	
76	(2*S*)-7-methoxy-6- (2-hydroxy-3-methylbut-3-en-1-yl)-2-(4-hydroxyphenyl) chroman-4-one	$C_{21}H_{22}O_5$	354.40	(Yang et al., 2024)
77	(2*S*)-6-[(2″*R*)-2″,3″-dihydroxy-3″-methylbutyl]-2-(4-hydroxyphenyl)-7-methoxyflavanone	$C_{21}H_{24}O_6$	372.42	
78	brosimacutin D	$C_{20}H_{20}O_5$	340.4	(Liu, 2019)
79	brosimacutin E	$C_{20}H_{20}O_5$	340.4	
80	bavadin	$C_{27}H_{30}O_{13}$	562.1686	(Yang and Qin, 2006)
81	8-geranyldaidzein	$C_{24}H_{24}O_4$	376.45	(Liu et al., 2021)
82	bavaisoflavone A	$C_{17}H_{10}O_4$	278.26	
83	bavaisoflavone B	$C_{20}H_{14}O_4$	318.33	
84	bavaisoflavone C	$C_{20}H_{16}O_5$	336.34	
85	bavaisoflavone D	$C_{21}H_{18}O_5$	350.37	
86	bavaisoflavone E	$C_{20}H_{18}O_6$	354.36	
87	bavaisoflavone F	$C_{20}H_{18}O_5$	338.36	
88	bavaisoflavone G	$C_{20}H_{18}O_5$	338.36	
89	bavaisoflavone H	$C_{23}H_{24}O_7$	412.44	(Xu et al., 2022)
90	bavaisoflavone I	$C_{25}H_{28}O_6$	424.49	
91	bavaisoflavone J	$C_{25}H_{26}O_6$	422.48	
92	bavaisoflavone K	$C_{25}H_{26}O_5$	406.48	
93	bavaisoflavone L	$C_{25}H_{26}O_6$	422.48	
94	bavaisoflavone M	$C_{25}H_{26}O_6$	422.48	
95	bavaisoflavone N	$C_{25}H_{24}O_5$	404.46	
96	bavaisoflavone O	$C_{25}H_{24}O_5$	404.46	
97	bavaisoflavone P	$C_{25}H_{26}O_5$	406.48	

98	corylifol A	$C_{25}H_{26}O_4$	390.1831	(Yin et al., 2004)
99	corylifols D	$C_{20}H_{18}O_5$	338.36	(Song et al., 2013)
100	corylifols E	$C_{20}H_{18}O_6$	354.1103	
101	corylifol G	$C_{25}H_{26}O_5$	406.4	(Du et al., 2019)
102	neobavaisoflavone	$C_{20}H_{18}O_4$	322.4	(Li, 2019)
103	isoneobavaisoflavone	$C_{20}H_{18}O_4$	322.4	(Yin et al., 2004)
104	corylin	$C_{20}H_{16}O_4$	320.3	(Liu, 2019)
105	genistein	$C_{15}H_{10}O_5$	270.0528	
106	daidzein	$C_{15}H_{10}O_4$	254.0579	(Hsu et al., 2001)
107	biochanin A	$C_{16}H_{11}O_5$	284.0685	
108	8-prenyldaidzein	$C_{20}H_{18}O_4$	322.1205	(Qiu et al., 2010)
109	bakuisoflavone	$C_{20}H_{18}O_5$	338.10	(Cui et al., 2015)
110	erythrinin A	$C_{20}H_{16}O_4$	320.1049	(Yin et al., 2004)
111	hydroxypsoralenol A	$C_{20}H_{18}O_5$	338.10	
112	hydroxypsoralenol B	$C_{20}H_{18}O_6$	354.36	(Suri et al., 1978)
113	psoralenol	$C_{20}H_{18}O_5$	338.1154	
114	neocorylin	$C_{25}H_{24}O_4$	322.1205	(Choi et al., 2008)
115	myrsininone A	$C_{25}H_{26}O_5$	406.48	
116	wighteone	$C_{20}H_{18}O_5$	338.1154	(Limper et al., 2013)
117	isowighteone	$C_{20}H_{18}O_5$	338.1154	
118	3′-hydroxydaidzein	$C_{15}H_{10}O_5$	270.24	
119	3′-methoxydaidzein	$C_{16}H_{12}O_5$	284.27	(Yang et al., 2024)
120	4′,7-dihydroxy-3′,5′-diprenyl-isoflavone	$C_{25}H_{26}O_4$	390.48	
121	corylinal	$C_{16}H_{10}O_5$	282.0528	(Wei et al., 2019)
122	4′,7-dihydroxy-3′-(6″ β-hydroxy-3″,7″-dimethyl-,2″,7″-dibutenyl)-geranylisoflavone	$C_{25}H_{26}O_5$	406.48	(Lin et al., 2018)
123	4′,7-dihydroxy-3′-(7″-hydroxy-7″-methyl-2″,5″-dibutenyl)-geranylisoflavone	$C_{25}H_{26}O_5$	406.48	
124	daidzin	$C_{20}H_{18}O_9$	416.1107	(Qiu et al., 2010)
125	corylinin	$C_{25}H_{26}O_4$	390.5	(Ruan et al., 2007)
126	7-*O*-methylcorylifol A	$C_{26}H_{28}O_4$	404.51	
127	7-*O*-isoprenylcorylifol A	$C_{30}H_{34}O_4$	458.60	(Chen et al., 2017)
128	7-*O*-isoprenylneobavaisoflavone	$C_{25}H_{26}O_4$	390.48	
129	5,7,4'-trihydroxyflavone	$C_{15}H_{10}O_5$	270.0528	(Liu et al., 2008)
130	7-methoxybakuchiol	$C_{21}H_{20}O_5$	352.39	
131	3″-acetoxy-7-methoxybakuchiol	$C_{23}H_{22}O_6$	394.42	(Suri et al., 1978)
132	3″,7-acetoxybakuchiol	$C_{25}H_{25}O_7$	437.47	
133	corylisoflavone A	$C_{18}H_{18}O_6$	330.34	(Dong et al., 2015)
134	bavarigenin-4″-O-β-d-	$C_{27}H_{30}O_{12}$	561.1976	(Shi et al.,

	glucopyranoside			2024)
135	bavarigenin-4″-O-β-d-glucopyranoside-4′-O-β-d-fucopyranoside	$C_{33}H_{40}O_{16}$	705.2367	
136	daidzein-3′-carboxylic acid	$C_{16}H_{10}O_{6}$	297.0413	
137	bavachalcone	$C_{20}H_{20}O_{4}$	324.40	(Bhalla et al.,
138	isobavachalcone	$C_{20}H_{20}O_{4}$	324.40	1968)
139	neobavachalcone	$C_{17}H_{14}O_{6}$	314.0790	(Gupta et al., 1977)
140	isoneobavachalcone	$C_{17}H_{14}O_{5}$	314.0790	(Bhalla et al., 1968)
141	bavachromanol	$C_{20}H_{20}O_{5}$	340.4	(Suri et al., 1980)
142	corylifol B	$C_{20}H_{20}O_{5}$	340.4	(Yin et al., 2004)
143	bavachromene	$C_{20}H_{18}O_{4}$	322.1205	(Lee et al.,
144	isobavachromene	$C_{20}H_{18}O_{4}$	322.1205	2005)
145	brosimacutin G	$C_{20}H_{20}O_{6}$	356.4	(Yin et al., 2004)
146	bakuchalcone	$C_{20}H_{20}O_{5}$	340.4	
147	psorachalcone A	$C_{20}H_{20}O_{5}$	340.38	(Yu, 2005)
148	psorachalcone B	$C_{20}H_{20}O_{5}$	340.38	
149	psorachalcone C	$C_{21}H_{24}O_{7}$	388.42	(Xu et al., 2022)
150	psorachromene	$C_{20}H_{18}O_{4}$	322.36	
151	4,2′-dihydroxy-2″-(1‴-methylethyl)-2″3″-dihydro-(4″,5″,3′,4′) furano chalk one	$C_{20}H_{20}O_{4}$	324.1362	(Agarwal et al., 2006)
152	4′-*O*-methylbavachalcone	$C_{21}H_{22}O_{4}$	338.40	
153	xanthoangelol	$C_{25}H_{28}O_{4}$	392.1988	(Limper et al., 2013)
154	kanzonol B	$C_{25}H_{24}O_{4}$	388.46	
155	licoagrochalcone A	$C_{20}H_{20}O_{4}$	324.38	
156	lespecyrtin B_{3}	$C_{20}H_{20}O_{5}$	340.38	
157	(1- [2, 4-dihydroxy-3- (3-methylbut-2-enyl)phenyl] -3- (4-hydroxyphenyl)propan- 1-one)	$C_{20}H_{22}O_{4}$	326.39	
158	(*E*)-1-(6-hydroxybenzofuran-5-yl) -3-(4-hydroxyphenyl)prop-2-en-1-one	$C_{17}H_{12}O_{4}$	280.28	(Yang et al., 2024)
159	(*E*) -1- (4-hydroxybenzofuran-5-yl) -3-(4-hydroxyphenyl)prop-2-en-1-one	$C_{17}H_{12}O_{4}$	280.28	
160	[3′, 4′-(3-hydroxy-2, 2-dimethyldihydropyrano)-2′, 4-dihydroxychalcone	$C_{20}H_{20}O_{5}$	340.38	
161	1-(3,4-dihydro-5-hydroxy-2,2-dimethyl-2*H*-1-benzopyran-8-yl) -3- (4-hydroxyphenyl) -2-propen-1-one	$C_{20}H_{20}O_{4}$	324.38	
162	artonin ZA	$C_{20}H_{18}O_{4}$	332.36	(Du et al.,

				2019)
163	4,2',3'-trihydroxy-4'-(2"β-hydroxy-3"-methybult-3"-enyl) chalcone	$C_{20}H_{20}O_5$	340.38	
164	4,2',3'-trihydroxy-4'-(2"α-hydroxy-3"-methybult-3"-enyl) chalcone	$C_{20}H_{20}O_5$	340.38	
165	4,2'-dihydroxy-4'-methoxy-5'-(2"β-hydroxy-3"-methybult-3"-enyl) chalcone	$C_{21}H_{20}O_5$	354.40	
166	4,2',4'-trihydroxy-3'-(2"α-3"-dihydroxy-3"-dimethybult) chalcone	$C_{20}H_{22}O_6$	358.39	(Zhao, 2023)
167	4,2',4'-trihydroxy-3'-(2"β-3"-dihydroxy-3"-dimethybult) chalcone	$C_{20}H_{22}O_6$	358.39	
168	4-hydroxy-3',4'-(2"α-3"-hydroxy-3"-dimethybult) furano-chalcone	$C_{20}H_{20}O_5$	340.38	
169	4,2',3'-trihydroxy-4'-methoxy-5'-(2"β-hydroxy-3"-methoxy-3"-dimethybult) chalcone	$C_{22}H_{26}O_6$	386.44	
170	4,2',3'-trihydroxy-4'-prenyl-chalcone	$C_{20}H_{20}O_4$	324.38	
171	4,2′-dihydroxy-4′-methoxy-5′-(3″,3″dimethylallyl) chalk one	$C_{21}H_{22}O_4$	354.40	(Gao et al., 2021)
172	bavaisoflavanol	$C_{20}H_{22}O_6$	358.39	(Xu et al., 2022)
173	6-(γ,γ-dimethylallyl)-4′-hydroxy-7-methoxy-(2R,3R)-dihydroflavonol	$C_{21}H_{22}O_5$	353.1400	(Shi et al., 2024)
174	coryaurone A	$C_{20}H_{18}O_6$	354.36	
175	(2*Z*) -2- [(4′-hydroxyphenyl) -methylene] -6-hydroxy-7-prenyl-3(2*H*) -benzofurane	$C_{20}H_{18}O_4$	322.36	
176	7,2′,5′-trihydroxy-8-prenylaurone	$C_{20}H_{18}O_5$	338.36	
177	licoagroaurone	$C_{20}H_{18}O_5$	338.36	
178	damaurone C	$C_{21}H_{18}O_5$	350.37	
179	damaurone D	$C_{20}H_{18}O_4$	322.36	
180	(*Z*)-2-(4-methoxybenzylidene)- 7, 7-dimethyl-7, 8-dihydro-2*H*-furo[2, 3-*f*]chromene-3, 9-dione	$C_{22}H_{20}O_5$	364.40	(Zhao et al., 2021)
181	(*Z*) -4, 6-dimethoxy-7, 4′-dihydroxyaurone	$C_{17}H_{14}O_6$	314.29	
182	rugaurone B	$C_{16}H_{12}O_5$	284.27	
183	6,7,3′,4′-tetrahydroxyaurone	$C_{15}H_{10}O_6$	286.24	
184	(2*Z*) -2- [(3′, 4′-dihydroxyphenyl) -methylene] -6-methoxy-7-prenyl-3(*2H*) -benzofurane	$C_{21}H_{20}O_5$	352.39	
185	(2*Z*) -2- [(4′-hydroxyphenyl) -methylene] -6-methoxy-7-	$C_{21}H_{20}O_4$	336.39	

		prenyl-3 (2*H*)-benzofurane			
Meroterpenes	186	bakuchiol	$C_{18}H_{24}O$	256.1827	(Mehta et al., 1966)
	187	bisbakuchiols A	$C_{36}H_{46}O_4$	542.3396	(Wu et al., 2007)
	188	bisbakuchiols B	$C_{36}H_{46}O_4$	542.3396	
	189	bisbakuchiols C	$C_{36}H_{48}O_3$	528.78	(Wu et al., 2008)
	190	2,3-epoxybakuchiol	$C_{18}H_{24}O_2$	272.39	(Huang et al., 2014)
	191	delta(1),3-hydroxybakuchiol	$C_{18}H_{24}O_2$	272.1776	
	192	delta (3),2-hydroxybakuchiol	$C_{18}H_{24}O_2$	272.1776	
	193	corylifolin	$C_{13}H_{16}O$	188.1201	(Sun et al., 1998)
	194	psoracorylifol A	$C_{18}H_{24}O_3$	288.1725	(Yin et al., 2006)
	195	psoracorylifol B	$C_{18}H_{24}O_3$	288.1725	
	196	psoracorylifol C	$C_{18}H_{24}O_3$	288.1725	
	197	psoracorylifol D	$C_{18}H_{24}O_2$	272.1776	
	198	psoracorylifol E	$C_{18}H_{24}O_2$	272.1776	
	199	psoracorylifol F	$C_{18}H_{24}O_2$	272.1776	(Xiao et al., 2012)
	200	psoralen ether	$C_{23}H_{30}O$	324.51	(Yang et al., 2022)
	201	psoracorylifol G	$C_{19}H_{26}O$	270.42	
	202	psoracorylifol H	$C_{18}H_{24}O_3$	288.1725	
	203	7α,8α,12α-psoracorylifol A	$C_{19}H_{26}O_3$	302.41	(Zhao, 2023)
	204	7α,8α,12β-psoracorylifol A	$C_{19}H_{26}O_3$	302.41	
	205	7α,8β,12α-psoracorylifol A	$C_{19}H_{26}O_3$	302.41	
	206	(12′S)-bisbakuchiol C	$C_{36}H_{48}O_3$	528.3603	(Wu et al., 2008)
	207	12,13-dihydro-12,13-dihydroxybakuchiol	$C_{18}H_{26}O_3$	290.40	
	208	delta(1),3-bakuchiol	$C_{18}H_{22}O$	254.37	
	209	12,13-dihydro-13-hydroxybakuchiol	$C_{18}H_{26}O_2$	274.1933	(Huang et al., 2014)
	210	15-demetyl-12,13-dihydro-13-ketobakuchiol	$C_{17}H_{22}O_2$	258.36	
	211	delta(10)-12,13-dihydro-12(R)-methoxyisobakuchiol	$C_{19}H_{26}O_2$	286.1933	
	212	delta(10)-12,13-dihydro-12(S)-methoxyisobakuchiol	$C_{19}H_{26}O_2$	286.1933	
	213	delta(11)-12-hydroxy-12-dimethyl bakuchiol	$C_{18}H_{24}O_2$	272.39	(Lin et al., 2018)
	214	13-methoxyisobakuchiol	$C_{19}H_{26}O_2$	286.1933	(Huang et al., 2014b)
	215	13-ethoxyisobakuchiol	$C_{20}H_{28}O_2$	300.2089	
	216	13-hydroxyisobakuchiol	$C_{18}H_{24}O_2$	272.39	(Yang et al., 2024)
	217	8*α*-hydroxy-cyclobakuchiol C	$C_{18}H_{26}O_3$	290.1882	
	218	8-ketone-cyclobakuchiol C	$C_{18}H_{24}O_3$	288.1725	(Xiu et al., 2021)
	219	7*α*,8*β*-hydroxy-12*β*-cyclobakuchiol C	$C_{18}H_{26}O_3$	290.1882	
	220	12*α*-psoracorylifol F	$C_{18}H_{24}O_2$	272.1776	
	221	7*β*,8*α*-hydroxy-12*β*-psoracorylifol F	$C_{18}H_{24}O_2$	272.1776	
	222	psoracorylifether	$C_{23}H_{30}O$	322.49	(Yang et al., 2022)
	223	12R,13-diolbakuchiol	$C_{18}H_{26}O_3$	290.40	(Liu, 2019)
	224	12S,13-diolbakuchiol	$C_{18}H_{26}O_3$	290.40	
	225	corypsoriol A	$C_{18}H_{24}O_2$	272.1776	(Yang et al., 2024)
	226	corypsoriol B	$C_{18}H_{24}O_2$	272.1776	

	227	corypsoriol C	$C_{18}H_{24}O_3$	288.1725	
	228	corypsoriol D	$C_{18}H_{22}O_3$	286.37	
	229	corypsoriol E	$C_{18}H_{22}O_3$	286.37	
	230	corypsoriol F	$C_{18}H_{24}O_4$	304.39	
	231	corypsoriol G	$C_{18}H_{24}O_4$	304.39	
	232	corypsoriol H	$C_{18}H_{24}O_2$	272.1776	
	233	corypsoriol I	$C_{19}H_{26}O_3$	302.41	
	234	corypsoriol J	$C_{19}H_{26}O_3$	302.41	
	235	corypsoriol K	$C_{19}H_{26}O_3$	302.41	
	236	corypsoriol L	$C_{19}H_{26}O_3$	302.41	
	237	corypsoriol M	$C_{19}H_{26}O_3$	302.41	
	238	corypsoriol N	$C_{19}H_{26}O_3$	302.41	
	239	cyclobakuchiol A	$C_{18}H_{24}O$	256.1827	(Huang et al., 2014)
	240	cyclobakuchiol B	$C_{18}H_{24}O$	256.1827	
	241	cyclobakuchiol C	$C_{18}H_{26}O_2$	274.1933	(Yin et al., 2007)
	242	bakuchiol ether A	$C_{29}H_{42}O_2$	422.65	(Xu et al., 2021)
	243	bakuchiol ether B	$C_{33}H_{50}O_2$	478.76	
	244	bakuchiol ether C	$C_{32}H_{46}O_2$	462.72	
	245	bisbakuchiol A	$C_{36}H_{46}O_4$	542.76	(Wu et al., 2007)
	246	bisbakuchiol B	$C_{36}H_{46}O_4$	542.76	
	247	bisbakuchiol D	$C_{36}H_{48}O_3$	528.78	(Xu et al., 2021)
	248	bisbakuchiol E	$C_{36}H_{48}O_3$	528.78	
	249	bisbakuchiol F	$C_{36}H_{48}O_3$	528.78	
	250	bisbakuchiol G	$C_{36}H_{48}O_3$	528.78	
	251	bisbakuchiol H	$C_{36}H_{46}O_4$	542.76	
	252	bisbakuchiol I	$C_{36}H_{46}O_4$	542.76	
	253	bisbakuchiol J	$C_{36}H_{48}O_4$	544.78	
	254	bisbakuchiol K	$C_{36}H_{48}O_4$	544.78	
	255	bisbakuchiol L	$C_{36}H_{48}O_4$	544.78	
	256	bisbakuchiol V	$C_{36}H_{48}O_4$	544.78	(Yang et al., 2022)
	257	bisbakuchiol M	$C_{36}H_{40}O_4$	536.71	(Xu et al., 2021)
	258	bisbakuchiol N	$C_{36}H_{46}O_2$	510.76	
	259	bisbakuchiol O	$C_{36}H_{46}O_2$	510.76	
	260	bisbakuchiol P	$C_{36}H_{46}O_2$	510.76	
	261	bisbakuchiol Q	$C_{36}H_{46}O_3$	526.76	
	262	bisbakuchiol R	$C_{37}H_{50}O_4$	558.80	
	263	bisbakuchiol S	$C_{37}H_{50}O_4$	558.80	
	264	bisbakuchiol T	$C_{36}H_{46}O_3$	526.76	
	265	bisbakuchiol U	$C_{36}H_{46}O_3$	526.76	
	266	12′*S*-bisbakuchiol C	$C_{36}H_{48}O_3$	528.78	(Yin et al., 2007)
Benzofurans	267	corylifonol	$C_{13}H_{14}O_4$	234.25	(Kuo and Lin, 1992)
	268	isocorylifonol	$C_{13}H_{14}O_4$	234.25	
	269	dihydrobutylcnideoside A	$C_{21}H_{28}O_9$	424.45	(He et al., 2021)
	270	butylcnideoside A	$C_{21}H_{26}O_9$	422.43	
	271	methylcnidioside A	$C_{18}H_{20}O_9$	380.35	
	272	isopsoralenoside butyl ester	$C_{21}H_{26}O_9$	422.43	
	273	isopsoralenoside methyl ester	$C_{18}H_{20}O_9$	380.35	
	274	dihydroisopsoralenoside butyl ester	$C_{21}H_{28}O_9$	424.45	
	275	dihydroisopsoralenoside methyl ester	$C_{18}H_{22}O_9$	382.37	
Dimers	276	psocorylin A	$C_{39}H_{46}O_6$	610.79	(Xu et al., 2020)
	277	psocorylin B	$C_{38}H_{44}O_6$	596.76	

	278	psocorylin C	$C_{38}H_{44}O_6$	596.76	
	279	psocorylin D	$C_{38}H_{44}O_6$	596.76	
	280	psocorylin E	$C_{38}H_{44}O_6$	596.76	
	281	psocorylin F	$C_{38}H_{42}O_6$	594.75	
	282	psocorylin G	$C_{39}H_{44}O_6$	608.78	
	283	psocorylin H	$C_{39}H_{44}O_5$	592.78	
	284	psocorylin I	$C_{39}H_{44}O_6$	608.78	
	285	psocorylin J	$C_{39}H_{46}O_6$	610.79	
	286	psocorylin K	$C_{39}H_{46}O_7$	626.79	
	287	psocorylin L	$C_{39}H_{44}O_5$	592.78	
	288	psocorylin M	$C_{38}H_{44}O_6$	596.76	
	289	psocorylin N	$C_{38}H_{42}O_7$	610.75	
	290	psocorylin O	$C_{38}H_{42}O_7$	610.75	
	291	psocorylin P	$C_{38}H_{38}O_7$	606.72	
	292	psocorylin Q	$C_{38}H_{40}O_5$	576.73	
	293	psocorylin R	$C_{40}H_{40}O_8$	648.75	
	294	psocorylin S	$C_{40}H_{40}O_8$	648.75	
	295	psocorylin T	$C_{40}H_{38}O_9$	662.74	
	296	psocorylin U	$C_{40}H_{38}O_9$	662.74	
	297	psocorylin V	$C_{40}H_{36}O_9$	660.72	(Xu et al., 2023)
	298	psocorylin W	$C_{40}H_{36}O_9$	660.72	
	299	psocorylin X	$C_{40}H_{36}O_{10}$	676.72	
	300	psocorylin Y	$C_{39}H_{40}O_9$	652.74	
	301	psocorylin Z	$C_{41}H_{36}O_9$	672.73	
	302	uracil	$C_4H_4N_2O_2$	112.09	(Ruan and Kong, 2005)
	303	β-stigmasterol	$C_{29}H_{48}O$	412.70	(Tan, 2017)
	304	psoralester	$C_{22}H_{38}O_4$	366.54	(Tewari and Bhakuni, 2010)
	305	palmitic acid	$C_{16}H_{32}O_2$	256.43	
	306	stearic acid	$C_{18}H_{36}O_2$	284.48	(Tan, 2017)
	307	linoleic acid	$C_{18}H_{32}O_2$	280.45	
	308	pinitol	$C_7H_{14}O_6$	194.18	(Qiu et al., 2010)
	309	daucosterol	$C_{35}H_{60}O_6$	576.86	
	310	triacylglycerols	$C_{39}H_{74}O_6$	639.02	
	311	glycerol diesters	$C_{27}H_{52}O_5$	456.71	(Lu et al., 2019)
Other metabolites	312	glycerol monoesters	$C_{21}H_{44}O_3$	344.58	
	313	raffinose	$C_{11}H_{20}O_{10}$	312.27	
	314	methyl p-hydroxybenzoate	$C_8H_8O_3$	152.15	(Qiu et al., 2010)
	315	p-hydroxybenzaldehyde	$C_7H_6O_2$	122.12	(Peng et al., 1996)
	316	adenosine	$C_{10}H_{13}N_5O_4$	267.25	
	317	guanosine	$C_{10}H_{13}N_5O_5$	283.24	(He et al., 2021)
	318	1-ribitol-2, 3-dione-1, 2, 3, 4-tetrahydro-6, 7-dimethyl-quinoxaline	$C_{15}H_{20}N_2O_6$	324.33	
	319	glyinflain A	$C_{25}H_{28}O_5$	408.49	(Zhao et al., 2021)
	320	stigmasterol-3-*O*-*β*-*D*-glucopyranosyl-6′-*O*-palmitate	$C_{51}H_{88}O_7$	813.26	(Yang et al., 2022)
	321	*p*-hydroxybenzaldehyde	$C_7H_6O_2$	122.12	

REFERENCES

Agarwal, D., Garg, S. P., and Sah, P. (2006). Isolation of chalkones from the seeds of *Psoralea corylifolia* Linn. doi:10.1002/chin.200711198

Bhalla, V. K., Nayak, U. R., and Dev, S. (1968). Some new flavonoids from Psoralea corylifolia. *Tetrahedron Letters* 9, 2401–2406.

Chai, M.-Y. (2020). A new bioactive coumestan from the seeds of Psoralea corylifolia. *J Asian Nat Prod Res* 22, 295–301. doi: 10.1080/10286020.2018.1563073

Chen, C.-H., Hwang, T.-L., Chen, L.-C., Chang, T.-H., Wei, C.-S., and Chen, J.-J. (2017). Isoflavones and anti-inflammatory constituents from the fruits of Psoralea corylifolia. *Phytochemistry* 143, 186–193. doi: 10.1016/j.phytochem.2017.08.004

Choi, Y. H., Yon, G. H., Hong, K. S., Yoo, D. S., Choi, C. W., Park, W.-K., et al. (2008). In vitro BACE-1 inhibitory phenolic components from the seeds of Psoralea corylifolia. *Planta Med* 74, 1405–1408. doi: 10.1055/s-2008-1081301

Cui, Y., Taniguchi, S., Kuroda, T., and Hatano, T. (2015). Constituents of Psoralea corylifolia Fruits and Their Effects on Methicillin-Resistant Staphylococcus aureus. *Molecules* 20, 12500–12511. doi: 10.3390/molecules200712500

Dong W., Wang Y., Zhou K., Liu G., Li L., Lou J., et al. (2015). A new compound of isoflavanones from fruits of Psoralea corylifolia and its cytotoxicity. *Chinese Traditional and Herbal Drugs* 46, 2206–2208. doi: 10.7501/j.issn.0253-2670.2015.15.004

Du, J., Wang, C.-H., Yang, J., He, X., Han, X.-L., Li, C.-C., et al. (2019). Chemical constituents from the fruits of Psoralea corylifolia and their protective effects on ionising radiation injury. *Nat Prod Res* 33, 673–680. doi: 10.1080/14786419.2017.1405407

Gao H., Gao P., Zang Y., Ma J., Yang J., Ye F., et al. (2021). Chemical Constituents of *Psoralea corylifolia* L. *Chinese Journal of Pharmacovigilance* 18, 556–561. doi: 10.19803/j.1672-8629.2021.06.13

Gupta, B. K., Gupta, G. K., Dhar, K. L., and Atal, C. K. (1980). Psoralidin oxide, a coumestan from the seeds of Psoralea corylifolia. *Phytochemistry* 19, 2232–2233. doi: 10.1016/s0031-9422(00)82240-2

Gupta, G. K., Dhar, K. L., and Atal, C. K. (1977). Isolation and constitution of corylidin: a new coumestrol from the fruits of Psoralea corylifolia. *Phytochemistry* 16, 403–404.

Gupta, S. R., Seshadri, T. R., and Sood, G. R. (1977). The structure and synthesis of neobavachalcone, a new component of Psoralea corylifolia. *Phytochemistry* 16, 1995–1997. doi: 10.1016/0031-9422(77)80111-8

Gupta, S., Jha, B. N., Gupta, G. K., Gupta, B. K., and Dhar, K. L. (1990). Coumestans from seeds of Psoralea corylifolia. *Phytochemistry* 29, 2371–2373. doi: 10.1016/0031-9422(90)83082-c

He, Z.-C., Xu, Q.-X., Yang, X.-W., Wang, Z.-J., and Xu, W. (2021). The benzofuran glycosides from the fruits of *Psoralea corylifolia* L. *Fitoterapia* 155, 105057. doi: 10.1016/j.fitote.2021.105057

Hsu, Y. T., Wu, C. J., Chen, J. M., Yang, Y. C., and Wang, S. Y. (2001). The presence of three isoflavonoid compounds in Psoralea corylifolia. *J Chromatogr Sci* 39, 441–

444. doi: 10.1093/chromsci/39.10.441
Huang S., Huang M., Jia X., and Hong R. (2014). Chemistry and Biology of Bakuchiol. *Chinese Journal of Organic Chemistry* 34, 2412–2423. doi: 10.6023/cjoc201408015
Huang, Y., Liu, X., Wu, Y., Li, Y., and Guo, F. (2014). Meroterpenes from Psoralea corylifolia against Pyricularia oryzae. *Planta Med* 80, 1298–1303. doi: 10.1055/s-0034-1382995
Ji L., and Xu Z. (1995). Review on Chemical Constituents of Psoralea corylifolia. *China Journal of Chinese Materia Medica*, 120-122+128.
Khastgir, H. N., Duttagupta, P. C., and Sengupta, P. (1961). The structure of psoralidin. *Tetrahedron* 14, 275–283. doi: 10.1016/S0040-4020(01)92176-3
Khatune, N. A., Islam, M. E., Haque, M. E., Khondkar, P., and Rahman, M. M. (2004). Antibacterial compounds from the seeds of Psoralea corylifolia. *Fitoterapia* 75, 228–230. doi: 10.1016/j.fitote.2003.12.018
Krishnamurti, M., and Parthasarathi, J. (1981). New syntheses of 4',7-dihydroxy-6,8-di-C-prenylflavanone, bavachin, isobavachin and related-compounds. *INDIAN J CHEM B* 20, 247–248.
Kulikov, O. A., Ageev, V. P., Brodovskaya, E. P., Shlyapkina, V. I., Petrov, P. S., Zharkov, M. N., et al. (2022). Evaluation of photocytotoxicity liposomal form of furanocoumarins Sosnowsky's hogweed. *Chem Biol Interact* 357, 109880. doi: 10.1016/j.cbi.2022.109880
Kuo, Y.-H., and Lin, Y.-L. (1992). Two new benzofuran derivatives, corylifonol and isocorylifonol from the seeds of Psoralea corylifolia. *Heterocycles* 34, 1555. doi:10.3987/com-92-6050
Lee, M. H., Kim, J. Y., and Ryu, J.-H. (2005). Prenylflavones from Psoralea corylifolia inhibit nitric oxide synthase expression through the inhibition of I-kappaB-alpha degradation in activated microglial cells. *Biol Pharm Bull* 28, 2253–2257. doi: 10.1248/bpb.28.2253
Li H. (2019). Study on Inhibition of Protein Tyrosine Phosphatase 1B and Diglyceride Acyltransferase Active Components by Chinese Medicine *Psoralea corylifolia* L. beihua university.
Limper, C., Wang, Y., Ruhl, S., Wang, Z., Lou, Y., Totzke, F., et al. (2013). Compounds isolated from Psoralea corylifolia seeds inhibit protein kinase activity and induce apoptotic cell death in mammalian cells. *J Pharm Pharmacol* 65, 1393–1408. doi: 10.1111/jphp.12107
Lin, X., Li, B.-B., Zhang, L., Li, H.-Z., Meng, X., Jiang, Y.-Y., et al. (2018). Four new compounds isolated from Psoralea corylifolia and their diacylglycerol acyltransferase (DGAT) inhibitory activity. *Fitoterapia* 128, 130–134. doi: 10.1016/j.fitote.2018.05.004
Liu H. (2019). The Isolation, Determination of Compounds in The Seeds of Psoralea corylifolia and Their DGAT Inhibitory Activity. *Jilin University*.
Liu H., Bai Y., Chen Y., and Zhao Y. (2008). Studies on Chemical Constituents of Psoralea corylifolia. *China Journal of Chinese Materia Medica*, 1410–1412.
Liu, X., Yang, J., Yu, H., Zhang, J., Du, J., Wang, X., et al. (2021). Chemical

constituents from the fruits of Cullen corylifolium (L.) Medik. by the targeted separation mode. *Nat Prod Res* 35, 1071–1076. doi: 10.1080/14786419.2019.1638382

Liu, Y., Dong, J.-Y., and Ren, B. (2018). A New Flavanone from Seeds of Psoralea corylifolia with α-Glucosidase Inhibitory Activity. *Natural Product Communications* 13, 1934578X1801300713. doi: 10.1177/1934578X1801300713

Lu Y., Zhang X., Wang J., Li K., Zhou N., and Zhang Z. (2019). Research Progress on Chemical Constituents and Pharmacological Actions of Psoralea Fructus. *Chinese Journal of Experimental Traditional Medical Formulae* 25, 180–189. doi: 10.13422/j.cnki.syfjx.20182022

Ma, S., Huang, Y., Zhao, Y., Du, G., Feng, L., Huang, C., et al. (2016). Prenylflavone derivatives from the seeds of Psoralea corylifolia exhibited PPAR-γ agonist activity. *Phytochemistry Letters* 16, 213–218. doi: 10.1016/j.phytol.2016.04.016

Matsuda, H., Sugimoto, S., Morikawa, T., Matsuhira, K., Mizuguchi, E., Nakamura, S., et al. (2007). Bioactive constituents from Chinese natural medicines. XX. Inhibitors of antigen-induced degranulation in RBL-2H3 cells from the seeds of Psoralea corylifolia. *Chem Pharm Bull (Tokyo)* 55, 106–110. doi: 10.1248/cpb.55.106

Mehta, G., Nayak, U. R., and Dev, S. (1966). Bakuchiol, a novel monoterpenoid. *Tetrahedron Letters* 7, 4561–4567.

Peng, G., Wu, P., Li, H., and Chen, D. (1996). Neo-psoralen isolated from *Psoralea corylifolia* Linn. *Natural Product Research and Development* 8, 31–34. doi: 10.16333/j.1001-6880.1996.03.008

Qiao, C.-F., Han, Q.-B., Mo, S.-F., Song, J.-Z., Xu, L.-J., Chen, S.-L., et al. (2006). Psoralenoside and isopsoralenoside, two new benzofuran glycosides from Psoralea corylifolia. *Chem Pharm Bull (Tokyo)* 54, 714–716. doi: 10.1248/cpb.54.714

Qiu R., Li L., and Le W. (2010). Research progress on chemical constituents and pharmacological effects of Psoralea corylifolia. *Journal of Chinese Medicinal Materials* 33, 1656–1659. doi: 10.13863/j.issn1001-4454.2010.10.040

Rajendra Prasad, N., Anandi, C., Balasubramanian, S., and Pugalendi, K. V. (2004). Antidermatophytic activity of extracts from *Psoralea corylifolia* (Fabaceae) correlated with the presence of a flavonoid compound. *Journal of Ethnopharmacology* 91, 21–24. doi: 10.1016/j.jep.2003.11.010

Ruan B., and Kong L. (2005). Studies on Chemical Constituents of Psoralea corylifolia. *Research & Information on Traditional Chinese Medicine*, 7–9.

Ruan, B., Kong, L.-Y., Takaya, Y., and Niwa, M. (2007). Studies on the chemical constituents of *Psoralea corylifolia* L. *J Asian Nat Prod Res* 9, 41–44. doi: 10.1080/10286020500289618

Shi, P., Wang, L., Qiu, X., Yu, X., Hayakawa, Y., Han, N., et al. (2024). The flavonoids from the fruits of Psoralea corylifolia and their potential in inhibiting metastasis of human non-small cell lung cancers. *Bioorg Chem* 150, 107604. doi: 10.1016/j.bioorg.2024.107604

Song, P., Yang, X.-Z., and Yuan, J.-Q. (2013). Cytotoxic constituents from Psoralea

corylifolia. *J Asian Nat Prod Res* 15, 624–630. doi: 10.1080/10286020.2013.793181

Srinivasan, S., and Sarada, D. V. L. (2012). Antifungal activity of phenyl derivative of pyranocoumarin from *Psoralea corylifolia* L. seeds by inhibition of acetylation activity of trichothecene 3-o-acetyltransferase (Tri101). *J Biomed Biotechnol* 2012, 310850. doi: 10.1155/2012/310850

Sun, N. J., Woo, S. H., Cassady, J. M., and Snapka, R. M. (1998). DNA polymerase and topoisomerase II inhibitors from Psoralea corylifolia. *Journal of natural products* 61, 362–366. doi: 10.1021/np970488q

Suri, J. L., Gupta, G. K., Dhar, K. L., and Atal, C. K. (1978). Psoralenol: a new isoflavone from the seeds of Psoralea corylifolia. *Phytochemistry* 17, 2046.

Suri, J. L., Gupta, G. K., Dhar, K. L., and Atal, C. K. (1980). Bavachromanol: a new chalcone from the seeds of Psoralea corylifolia. *Phytochemistry* 19, 336–337. doi: 10.1016/s0031-9422(00)819

Takashima, J., and Ohsaki, A. (2002). Brosimacutins A-I, nine new flavonoids from Brosimum acutifolium. *J Nat Prod* 65, 1843–1847. doi: 10.1021/np020241f

Tan W. (2017). Study on chemical constituents and pharmacological action of the Fructus Psoraleae. Donghua University.

Tewari, A., and Bhakuni, R. S. (2010). New constituents from Psoralea corylifolia. *Indian Journal of Chemistry - Section B Organic and Medicinal Chemistry* 49, 256–259.

Wei M., Wang S., Yang W., Li Y., and Li C. (2019). Chemical Constituents of Psoraleae Fructus and Its Main Toxic Ingredients. *Chinese Journal of Experimental Traditional Medical Formulae* 25, 207–219. doi: 10.13422/j.cnki.syfjx.20190517

Wu, C.-Z., Cai, X. F., Dat, N. T., Hong, S. S., Han, A.-R., Seo, E.-K., et al. (2007). Bisbakuchiols A and B, novel dimeric meroterpenoids from Psoralea corylifolia. *Tetrahedron Letters* 48, 8861–8864. doi: 10.1016/j.tetlet.2007.10.059

Wu, C.-Z., Hong, S. S., Cai, X. F., Dat, N. T., Nan, J.-X., Hwang, B. Y., et al. (2008). Hypoxia-inducible factor-1 and nuclear factor-κB inhibitory meroterpene analogues of bakuchiol, a constituent of the seeds of Psoralea corylifolia. *Bioorganic & medicinal chemistry letters* 18, 2619–2623. doi: 10.1016/j.bmcl.2008.03.028

Xiao, G., Li, X., Wu, T., Cheng, Z., Tang, Q., and Zhang, T. (2012). Isolation of a new meroterpene and inhibitors of nitric oxide production from Psoralea corylifolia fruits guided by TLC bioautography. *Fitoterapia* 83, 1553–1557. doi: 10.1016/j.fitote.2012.08.024

Xiu, M.-X., Zhao, Y.-M., Zhang, Y., Xiong, D.-X., Wang, D., Lee, H.-S., et al. (2021). Diacylglycerol acyltransferase inhibitory new meroterpenes from the seeds of Psoralea corylifolia, and their structure-activity relationship study. *Fitoterapia* 151, 104881. doi: 10.1016/j.fitote.2021.104881

Xu, D.-S., Zhao, Q., Wang, B., Wu, M., and Xu, J. (2021). Chemical constituents from the bioactive fraction of the seeds of Psoralea corylifolia and proliferation activities on osteoblastic-like UMR106 cells. *J Asian Nat Prod Res* 23, 975–981.

doi: 10.1080/10286020.2020.1803292

Xu, Q., Lv, Q., Liu, L., Zhang, Y., and Yang, X. (2021). New bakuchiol dimers from Psoraleae Fructus and their inhibitory activities on nitric oxide production. *Chin Med* 16, 98. doi: 10.1186/s13020-021-00499-y

Xu, Q., Zhang, Y., He, Z., Liu, Z., Zhang, Y., Xu, W., et al. (2022). Constituents promoting osteogenesis from the fruits of Psoralea corylifolia and their structure-activity relationship study. *Phytochemistry* 196, 113085. doi: 10.1016/j.phytochem.2022.113085

Xu, Q.-X., Wang, Z.-J., He, Z.-C., Xu, J., Xu, W., and Yang, X.-W. (2023). Flavonoids dimers from the fruits of Psoralea corylifolia and their cytotoxicity against MCF-7 cells. *Bioorg Chem* 130, 106262. doi: 10.1016/j.bioorg.2022.106262

Yadava, R. N., and Verma, V. (2005). A new biologically active flavonol glycoside from *Psoralea corylifolia* (Linn.). *J Asian Nat Prod Res* 7, 671–675. doi: 10.1080/10286020310001608921

Yang T., and Qin M. (2006). Isolation and structural identification of a new isoflavone from *Psoralea corylifolia* L. *Acta Pharmaceutica Sinica*, 76–79. doi: 10.16438/j.0513-4870.2006.01.014

Yang T., Li J., Qin M., Zhao M., Ouyang Z., and Fu H. (2009). Two new compounds from *Psoralea corylifolia* L. *Acta Pharmaceutica Sinica* 44, 1387–1390. doi: 10.16438/j.0513-4870.2009.12.003

Yang X., Lv Q., Xu Q., Xu W., and Zhang Y. (2022). Study on chemical constituents from cyclohexane soluble part of Psoraleae Fructus. *Chinese Traditional and Herbal Drugs* 53, 3269–3279.

Yang X., Xu Q., Lv Q., He Z., Xu W., and Zhang Y. (2024). Research Progress in Chemical Constituents of Psoraleae Fructus. *Modern Chinese Medicine* 26, 733–748. doi: 10.13313/j.issn.1673-4890.20231221001

Yin, S., Fan, C.-Q., and Yue, J.-M. (2007). Cyclobakuchiol C, a new bakuchiol derivative from Psoralea coryllfolia. *J Asian Nat Prod Res* 9, 29–33. doi: 10.1080/10286020500289568

Yin, S., Fan, C.-Q., Dong, L., and Yue, J.-M. (2006). Psoracorylifols A–E, five novel compounds with activity against Helicobacter pylori from seeds of Psoralea corylifolia. *Tetrahedron* 62, 2569–2575. doi: 10.1016/j.tet.2005.12.041

Yin, S., Fan, C.-Q., Wang, Y., Dong, L., and Yue, J.-M. (2004). Antibacterial prenylflavone derivatives from Psoralea corylifolia, and their structure-activity relationship study. *Bioorg Med Chem* 12, 4387–4392. doi: 10.1016/j.bmc.2004.06.014

Yu L. (2005). Studies on chemical constituents of *Psoralea corylifolia* L., Aristolochia contorta and Valeriana jatamaisi Jones. Yunnan Normal University.

Zhang Y., Han G., Li W., and Luo Z. (2017). Determination of Six Furcoumarins in Psoralea corylifolia from Different Areas by HPLC. *Chinese Journal of Pharmaceuticals* 48, 896–899. doi: 10.16522/j.cnki.cjph.2017.06.020

Zhao Y. (2023). Studies on new compounds obtained from Psoralea corylifolia and their biological activities. beihua university. doi: 10.26928/d.cnki.gbhuu.2023.000048

Zhao, Y.-M., Xiu, M.-X., Wang, D., Zhang, Y., Zhang, X.-Y., Shao, X.-J., et al. (2021).

Flavonoids from the seeds of Psoralea corylifolia inhibit diacylglycerol acyltransferase. *Phytochemistry Letters* 44, 120–124. doi: 10.1016/j.phytol.2021.06.013

Zhu, G., Luo, Y., Xu, X., Zhang, H., and Zhu, M. (2019). Anti-diabetic compounds from the seeds of Psoralea corylifolia. *Fitoterapia* 139, 104373. doi: 10.1016/j.fitote.2019.104373
