# Supplementary material for: A review of Psoralea corylifolia L.: a valuable plant with profound biological significance
Source: Front Pharmacol. 2025 Jan 20;15:1521040. doi: 10.3389/fphar.2024.1521040 (PMC11788583; doi:10.3389/fphar.2024.1521040)
Supplement: Supplementary file 2 [file Table2.pdf]

Supplementary Table 2 The pharmacological effects of *Psoralea corylifolia* L.

Pharmacological activity	Tested substance	Model	Tested living system/ organ/cell	Time period of application	Dose range	Result	References
Anti-osteoporosis	psoralen		$CD4^{+}$	8 d	10μmol/L	The balance of $CD4^{+}CD25^{+}$Treg/Th17 in $CD4^{+}$T cells developed to $CD4^{+}CD25^{+}$Treg ↑ RANKL induced RAW264.7 to differentiate into osteoclasts and bone resorption↓	(Li et al., 2018)
	psoralidin		Bone marrow macrophages (BMMs)	4 d	0, 1, 10, 30 or 50 μM	TNF-α, RANK/OPG↓ inhibition of p38, JNK and ERK osteoclast differentiation ↓	(Kong et al., 2017)
	isopsoralen	Establishment of OP rat model by intramuscular injection of dexamethasone sodium injection.	Rat	12 weeks	25 mg/ kg/ d	Inhibit Axin2/ PPAR-γ signal pathway, bone marrow lipid metabolism↓, osteoblast differentiation ↑	(Wang et al., 2021b)
	neobavaisoflavone	Establishment of OP mouse model by double ovariectomy	Mice	6 weeks	30 mg/kg	Inhibition of NF-κB signaling pathway, osteoclast differentiation ↓	(Chen et al., 2020)
Regulate estrogen level	isopsoralen		ER(-) breast cancer T47D cells and ER(+) breast cancer MDA-MB231 cells	48h	1×10^{-6}mol/L, 1×10^{-7}mol/L	Promote cell proliferation, increase the division and proliferation index of ER(+) breast cancer cells, and increase the expression of estrogen-responsive genes through ER.	(Zhao et al., 2009)
	Ethanol extract	Ovariectomized rat model	Rat	6 weeks	0.25 %/ 0.5 %	By increasing alkaline phosphatase, Ca concentration,	(Lim et al., 2009)

					extracts of diets	serum E2 concentration and bone mineral density, and decreasing inorganic P level, the postmenopausal bone loss can be reduced.	
	bakuchiol		MCF-7 cells	72h	4, 7, and 10 μg/ml	Inducing ERβ expression and inhibiting ERα expression induced S-phase stop of MCF-7 and MDA-MB-231 cells, blocked the activation of Cdc2, induced apoptosis of MCF-7 cells and disturbed mitochondrial membrane potential.	(Li et al., 2016)
	Ethanol extract		Mice	14d	4.5, 9, 18g/ kg	Increase the absolute weight and uterine coefficient of the uterus of sexually immature mice, and increase the level of estrogen and androgen in serum.	(Li et al., 2012)
Anti-tumor	corylin		HCC	72h		It significantly inhibited the proliferation, migration and invasion of HCC cells, and inhibited epithelial-mesenchymal transition. Up-regulating long-chain noncoding RNA growth inhibitory specific transcript 5 (GAS5) of tumor suppressor and activating its downstream anticancer pathway.	(Chen et al., 2018a)
	Ethanol extract		HCT116, SW480, LoVo and HT-29 cells	48h	0, 25, 50, 100 μg/ml	Inhibition of cyclin D1 and CDK4 expression by proteasome degradation	(Park et al., 2016)
	bavachinin	Rat colon cancer	Rat	18 weeks	50, 100, 200	Promote the expression of p53,	(Zhao et al.,

		model induced by xylene (DMH and DSS)			mg/kg	so as to inhibit cell proliferation by increasing the apoptosis of cancer cells, thus reducing ACF and proliferative lesions.	2021)
	psoralen		mice	6 weeks	17.5 mg/kg	It can significantly reduce bone metastasis of breast cancer in mice and reduce tumor load. Inhibit the growth of breast cancer cells in metastatic microenvironment and regulate the functions of osteoblasts and osteoclasts in tumor-bearing mice.	(Wu et al., 2013)
Antibacterial	bavachinin		S.aureus bacterial strain USA300、Newman、ATCC29213	36h	0.25, 0.50, 1.00, 2.00, 4.00, 8.00, 16.00, 32.00 mg/L	Significantly inhibit the generation of STX, reduce the survival rate of USA300 in acid-base stress environment, inhibit the adhesion of USA300 to A549 cells, and reduce the pathogenicity of S.aureus.	(Ding et al., 2022)
Antioxidant	bakuchiol				12 μM	Inhibition of tyrosinase kinetics, inhibition of styrene and potassium self-oxidation, antioxidant activity is entirely derived from phenol OH, and the side chain will play a role by increasing its reactivity.	(Cariola et al., 2023)
	Water extract	Palmitate (PA)-induced cytotoxicity of PC12 cells	PC12 cells	48h	1, 10, 30, 50, 100 μg/ml	Increasing the expression of bcl-2 protein reduced the production of ROS induced by pa, increased the mRNA levels of antioxidant genes such as nuclear factor-like 2 and heme oxygenase-1, restored the expression of	(Lee et al., 2016)

						autophagy marker genes beclin-1 and p62, and reduced the apoptosis induced by pa.	
	corylin		HEK293T and HaCaT cells	48h	10^{-5}–100 μM	Reduce oxidative stress and increase the nuclear expression of Nrf2, stimulate Nrf2 to migrate into the nucleus, and increase the transfer of its target antioxidant gene together with KeaP1 to dissociate Nrf2.	(Li et al., 2022b)
	bavachin	db/db mice	Mice	6 weeks	10mg/kg	Decrease urinary microalbumin, urea nitrogen and inosine gap, reduce glomerular area and collagen deposition in kidney of db/db mice, reduce renal levels of fibrosis factor, fibrosis protein, COL1A1 and α-SMA, up-regulate protein expression of antioxidant enzymes in kidney tissue, and reduce the production of mitochondrial ROS.	(Park et al., 2023)
Anti-inflammatory	bakuchiol	LPS Stimulated BV-2 Mouse Microglia	BV-2 cells	48h	0, 1.25, 2.5, 5, or 10 μM	Regulate the expression of IL-6, iNOS and COX-2, inhibit the production of IL-6, NO and PGE2, and inhibit the inflammatory response of microglia activated by lps-induced p38 MAPK and ERK signaling pathways.	(Lim et al., 2019)
	PCL polysaccharide		RAW264.7 cells	48h	25, 50, 100, 200, 400 and 800 μg/mL	Increase the level of NO, up-regulate the expression of iNOS mRNA, up-regulate the expression of TNF-α and IL-6 mRNA, further increase the	(Wang et al., 2021a)

						expression and phagocytosis of TNF-α, IL-6 and ROS, and up-regulate the phosphorylation level of p65, p38, ERK and JNK proteins.	
	corylin		BV2 cells	24h	0 to 10 μM	Inhibit the expression of iNOS and COX-2, weaken the phosphorylation of ERK, JNK and p38, reduce the expression of NLRP3 and ASC, and inhibit the activation of caspase-1 and IL-1β in BV2 cells activated by LPS.	(Huang et al., 2018)
	psoralen	Periodontitis model	Rat	4 weeks	8 mg/kg/2 d	Reducing TNF-α and increasing IL-10 decreased the RANKL/OPG ratio of periodontitis rats.	(Liu et al., 2021)
Neuroprotective	prenylflavonoids	AD model	Mice	9 months	50 mg/kg day	Significantly reduce the level of β -amyloid peptide 42, inhibit the hyperphosphorylation of microtubule-associated protein Tau, induce Ser9 phosphorylation of glycogen synthase kinase 3β, and reduce the expression of pro-inflammatory cytokines TNF-α, IL-6 and IL-1β.	(Chen et al., 2018b)
	bavachinin	AD cell model induced by amyloid β-protein	PC12 cells	48h	1×10^{-6}mol • L^{-1}	Reduce the secretion of inflammatory factors, improve the activity of antioxidant enzymes and mitochondrial membrane potential.	(Yang et al., 2021)
	corylin		HT22, BV2 cells	48h	2.5, 5, 10, 20, 25, and	Inhibit the production of ROS and NO, significantly increase	(Wang et al., 2023)

					50 μM	the activities of CAT and SOD, promote GSH secretion, inhibit the decrease of MMP, up-regulate the expression of Nrf2 and HO-1 proteins, inhibit the secretion of inflammatory factors IL-1β, IL-6 and TNF-α, and can specifically inhibit the transfer of NF-κBp65 into the nucleus.	
	Ethanol extract		PC12 cells	48h	10, 50, and 100 μg/ml	Increase the ATP level and mitochondrial membrane potential of PC12 cells, and increase the oxygen consumption of cells.	(Im et al., 2014)
Activating blood	bakuchin		Rat		1μM	Activating endothelium-dependent NO-cGMP signaling pathway and inducing vasodilation.	(Li et al., 2011)
Antiasthma	Water extract	Asthmatic model	Rat	42d	2.5, 5.0, and 10.0g/kg/ day	Inhibit inflammatory infiltration and mucus secretion in lung tissue, reduce the expression of GATA-3 protein, and inhibit Th2 response.	(Jin et al., 2014)

REFERENCES

Cariola, A., El Chami, M., Granatieri, J., and Valgimigli, L. (2023). Anti-tyrosinase and antioxidant activity of meroterpene bakuchiol from Psoralea corylifolia (L.). Food Chem 405, 134953. doi: 10.1016/j.foodchem.2022.134953

Chen, C.-Y., Chen, C.-C., Shieh, T.-M., Hsueh, C., Wang, S.-H., Leu, Y.-L., et al. (2018a). Corylin Suppresses Hepatocellular Carcinoma Progression via the Inhibition of Epithelial-Mesenchymal Transition, Mediated by Long Noncoding RNA GAS5. *Int J Mol Sci* 19, 380. doi: 10.3390/ijms19020380

Chen, H., Fang, C., Zhi, X., Song, S., Gu, Y., Chen, X., et al. (2020). Neobavaisoflavone inhibits osteoclastogenesis through blocking RANKL

signalling-mediated TRAF6 and c-Src recruitment and NF-κB, MAPK and Akt pathways. J Cell Mol Med 24, 9067–9084. doi: 10.1111/jcmm.15543

Chen, Z.-J., Yang, Y.-F., Zhang, Y.-T., and Yang, D.-H. (2018b). Dietary Total Prenylflavonoids from the Fruits of *Psoralea corylifolia* L. Prevents Age-Related Cognitive Deficits and Down-Regulates Alzheimer's Markers in SAMP8 Mice. *Molecules* 23, 196. doi: 10.3390/molecules23010196

Ding Y., Zhu X., Li J., Liu S., Yu Q., and Wang L. (2022). Inhibitory effect of bavachinin on methicillin-resistant Staphylococcus aureus staphyloxanthin. *Chinese Journal of Veterinary Science* 42, 2514–2521. doi: 10.16303/j.cnki.1005-4545.2022.12.25

Huang, M.-Y., Tu, C.-E., Wang, S.-C., Hung, Y.-L., Su, C.-C., Fang, S.-H., et al. (2018). Corylin inhibits LPS-induced inflammatory response and attenuates the activation of NLRP3 inflammasome in microglia. *BMC Complement Altern Med* 18, 221. doi: 10.1186/s12906-018-2287-5

Im, A.-R., Chae, S.-W., Zhang, G. J., and Lee, M.-Y. (2014). Neuroprotective effects of *Psoralea corylifolia* Linn seed extracts on mitochondrial dysfunction induced by 3-nitropropionic acid. *BMC Complement Altern Med* 14, 370. doi: 10.1186/1472-6882-14-370

Jin, H., Wang, L., Xu, C., Li, B., Luo, Q., Wu, J., et al. (2014). Effects of Psoraleae fructus and its major component psoralen on Th2 response in allergic asthma. *Am J Chin Med* 42, 665–678. doi: 10.1142/S0192415X14500438

Kong, L., Ma, R., Yang, X., Zhu, Z., Guo, H., He, B., et al. (2017). Psoralidin suppresses osteoclastogenesis in BMMs and attenuates LPS-mediated osteolysis by inhibiting inflammatory cytokines. *Int Immunopharmacol* 51, 31–39. doi: 10.1016/j.intimp.2017.07.003

Lee, Y., Jun, H.-S., and Oh, Y. S. (2016). Protective Effect of *Psoralea corylifolia* L. Seed Extract against Palmitate-Induced Neuronal Apoptosis in PC12 Cells. *Evid Based Complement Alternat Med* 2016, 5410419. doi: 10.1155/2016/5410419

Li J., Xie B., Zhang W., Shi L., Li W., Zeng Y., et al. (2018). Psoralen inhibits RAW264.7 differentiation into osteoclasts and bone resorption by regulating CD4+T cell differentiation. *China Journal of Chinese Materia Medica* 43, 1228–1234. doi: 10.19540/j.cnki.cjcmm.20180104.017

Li, L., Chen, X., Liu, C. C., Lee, L. S., Man, C., and Cheng, S. H. (2016). Phytoestrogen Bakuchiol Exhibits In Vitro and In Vivo Anti-breast Cancer Effects by Inducing S Phase Arrest and Apoptosis. *Front Pharmacol* 7, 128. doi: 10.3389/fphar.2016.00128

Li L., Qiu R., Le W., Ju X., Fan N., Song H., et al. (2012). Study on Phytoestrogenic Effect in Psoralea Colrylifolia. *Journal of Liaoning University of Traditional Chinese Medicine* 14, 57–58. doi: 10.13194/j.jlunivtcm.2012.04.59.lil.097

Li, N., Liu, T., Zhu, S., Yang, Y., Wang, Z., Zhao, Z., et al. (2022b). Corylin from Psoralea fructus (*Psoralea corylifolia* L.) protects against UV-induced skin aging by activating Nrf2 defense mechanisms. *Phytother Res* 36, 3276–3294. doi: 10.1002/ptr.7501

Li, X., Lee, Y. J., Kim, Y. C., Jeong, G. S., Cui, H. Z., Kim, H. Y., et al. (2011). Bakuchicin induces vascular relaxation via endothelium-dependent

NO-cGMP signaling. *Phytother Res* 25, 1574–1578. doi: 10.1002/ptr.3478

Lim, H.-S., Kim, Y. J., Kim, B.-Y., and Jeong, S.-J. (2019). Bakuchiol Suppresses Inflammatory Responses Via the Downregulation of the p38 MAPK/ERK Signaling Pathway. *Int J Mol Sci* 20, 3574. doi: 10.3390/ijms20143574

Lim, S.-H., Ha, T.-Y., Kim, S.-R., Ahn, J., Park, H. J., and Kim, S. (2009). Ethanol extract of *Psoralea corylifolia* L. and its main constituent, bakuchiol, reduce bone loss in ovariectomised Sprague-Dawley rats. *Br J Nutr* 101, 1031–1039. doi: 10.1017/S0007114508066750

Liu, H., Xu, Y., Cui, Q., Liu, N., Chu, F., Cong, B., et al. (2021). Effect of Psoralen on the Intestinal Barrier and Alveolar Bone Loss in Rats With Chronic Periodontitis. *Inflammation* 44, 1843–1855. doi: 10.1007/s10753-021-01462-7

Park, G. H., Sung, J. H., Song, H. M., and Jeong, J. B. (2016). Anti-cancer activity of Psoralea fructus through the downregulation of cyclin D1 and CDK4 in human colorectal cancer cells. *BMC Complement Altern Med* 16, 373. doi: 10.1186/s12906-016-1364-x

Park, J., Seo, E., and Jun, H.-S. (2023). Bavachin alleviates diabetic nephropathy in db/db mice by inhibition of oxidative stress and improvement of mitochondria function. *Biomed Pharmacother* 161, 114479. doi: 10.1016/j.biopha.2023.114479

Wang, H., Xu, X., Yin, Z., Wang, M., Wang, B., Ma, C., et al. (2021a). Activation of RAW264.7 cells by PCp-I, a polysaccharide from *Psoralea corylifolia* L, through NF-κB/MAPK signalling pathway. *Int J Immunopathol Pharmacol* 35, 20587384211010058. doi: 10.1177/20587384211010058

Wang J., Wang J., Lu M., Mu T., and Geng Y. (2021b). Effects of Isopsoralen on Bone Metabolism in Rats Via PPAR Gamma-Axin 2-Wnt Signal Regulation. *World Chinese Medicine* 16, 1413–1416. doi: 10.3969/j.issn.1673-7202.2021.09.011

Wang, Z., Xu, Q., Xu, J., Xu, W., and Yang, X. (2023). Anti-oxidative and anti-neuroinflammatory effects of corylin in H2O2-induced HT22 cells and LPS-induced BV2 cells by activating Nrf2/HO-1 and inhibiting NF-κB pathways. *Journal of Chinese Pharmaceutical Sciences* 32, 85–100.

Wu, C., Sun, Z., Ye, Y., Han, X., Song, X., and Liu, S. (2013). Psoralen inhibits bone metastasis of breast cancer in mice. *Fitoterapia* 91, 205–210. doi: 10.1016/j.fitote.2013.09.005

Yang L., Li S., Wang Y., An L., Li J., Chen Q., et al. (2021). Protective Effect of Bavachinin A on PC12 Cells Injury Induced by Aβ and Its Mechanism. *Traditional Chinese Drug Research & Clinical Pharmacology* 32, 68–72. doi: 10.19378/j.issn.1003-9783.2021.01.009

Zhao, C., Ghosh, B., Chakraborty, T., and Roy, S. (2021). Bavachinin mitigates DMH induced colon cancer in rats by altering p53/Bcl2/BAX signaling associated with apoptosis. *Biotech Histochem* 96, 179–190. doi: 10.1080/10520295.2020.1778087

Zhao P., Niu J., Wang J., Hao Q., and Yu J. (2009). Study on phytoestrogenic effect of isopsoralen and its mechanism. *Chinese Pharmacological*

Bulletin 25, 1193–1197
