# Supplementary material for: A review of Psoralea corylifolia L.: a valuable plant with profound biological significance
Source: Front Pharmacol. 2025 Jan 20;15:1521040. doi: 10.3389/fphar.2024.1521040 (PMC11788583; doi:10.3389/fphar.2024.1521040)
Supplement: Supplementary file 3 [file DataSheet1.docx]

| 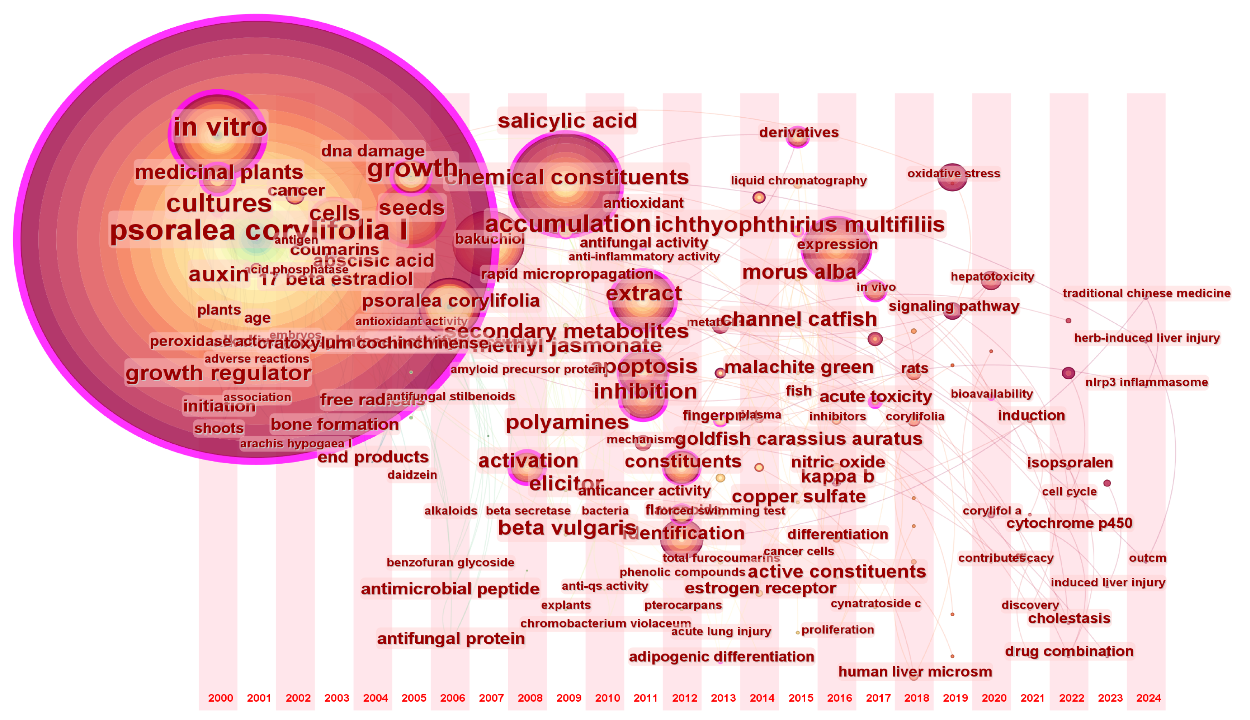 |
| --- |
| (A) |
| 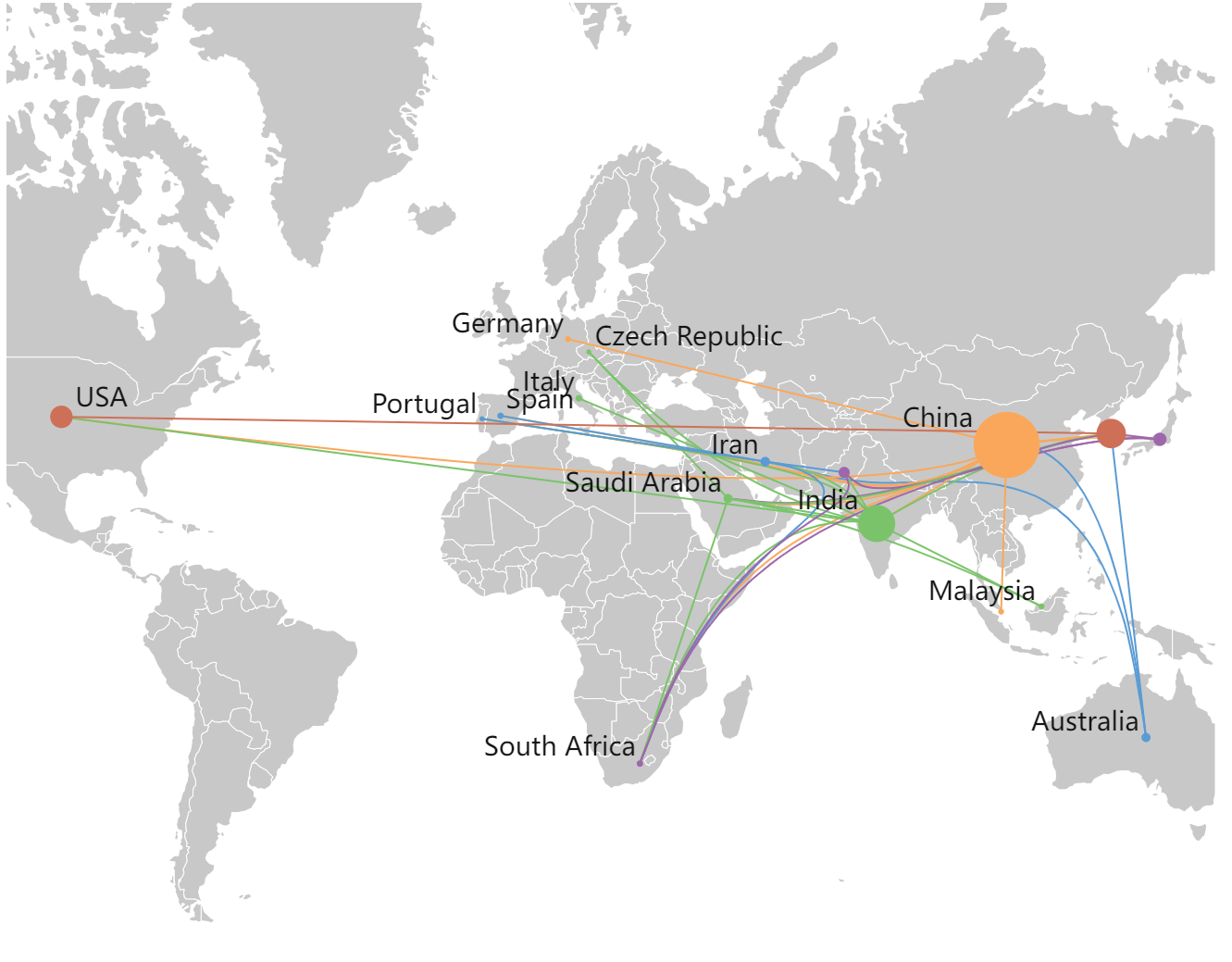 |
| (B) |

**Fig. S1** Time zone diagram of keywords in the research field of *Psoralea corylifolia* L. (A), Geographic visualization of the quantity of publications on *Psoralea corylifolia* L. (B).

| 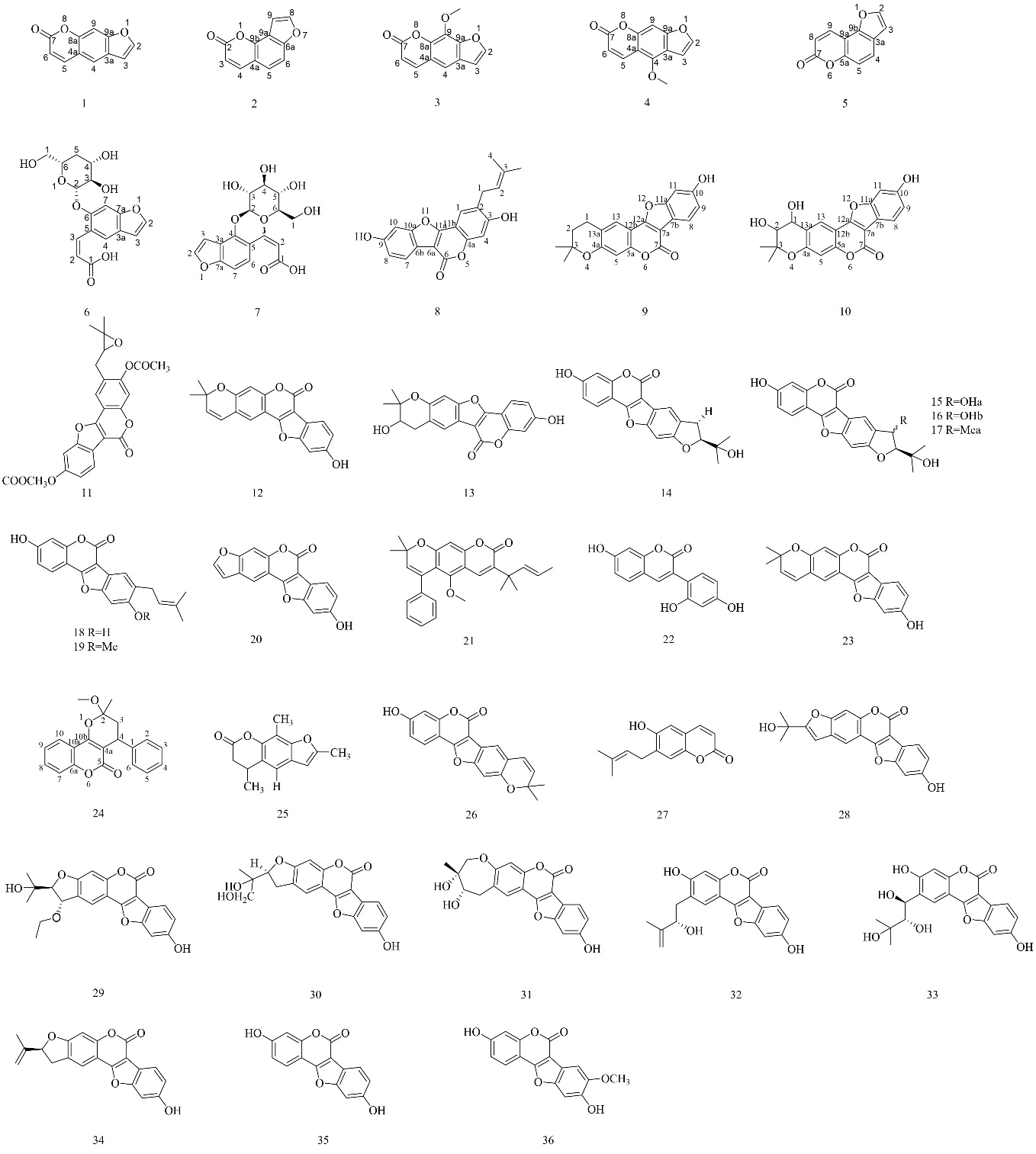 |
| --- |
| (A) |
| 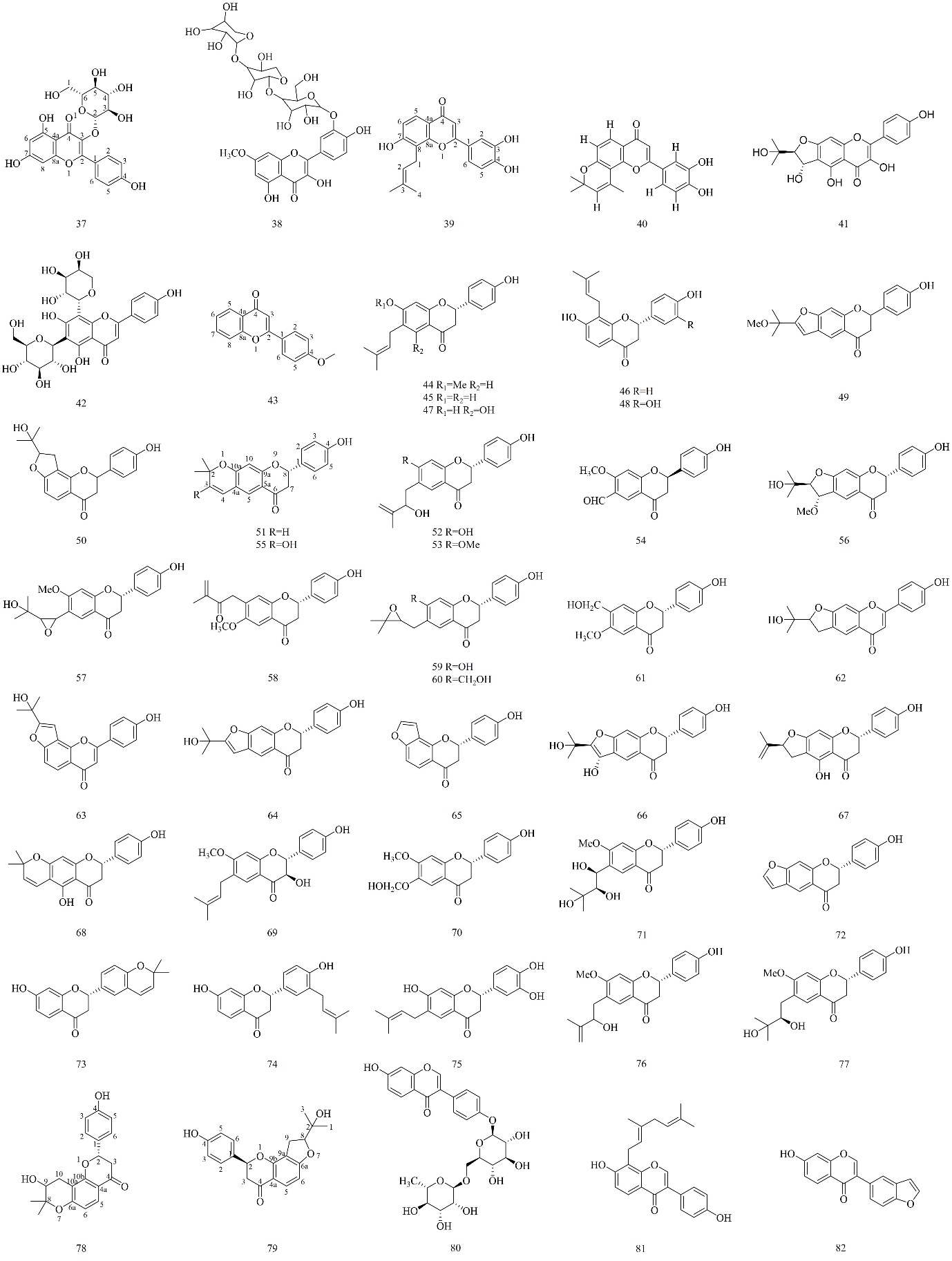 |
| (B) |
| 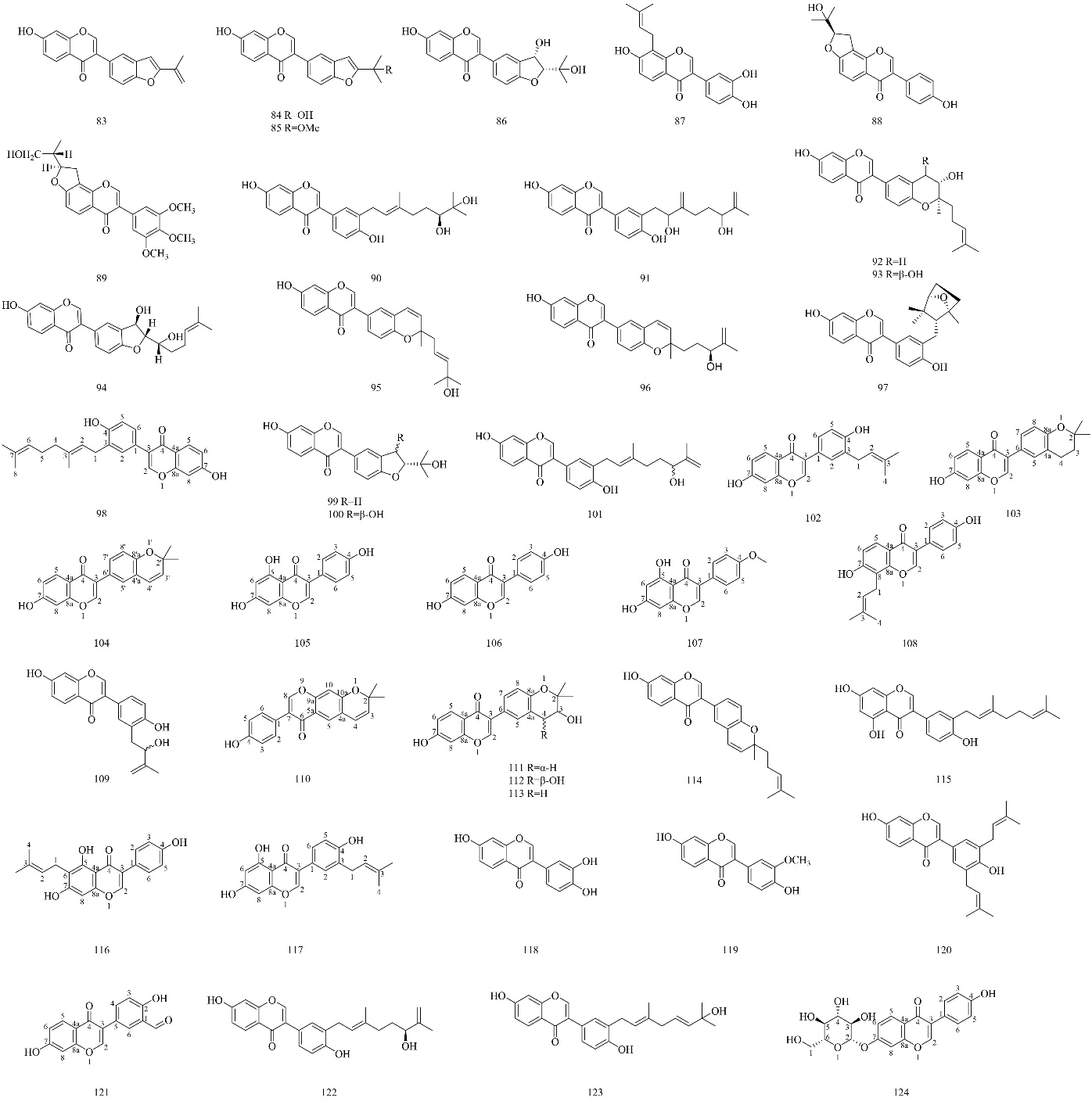 |
| (C) |
| 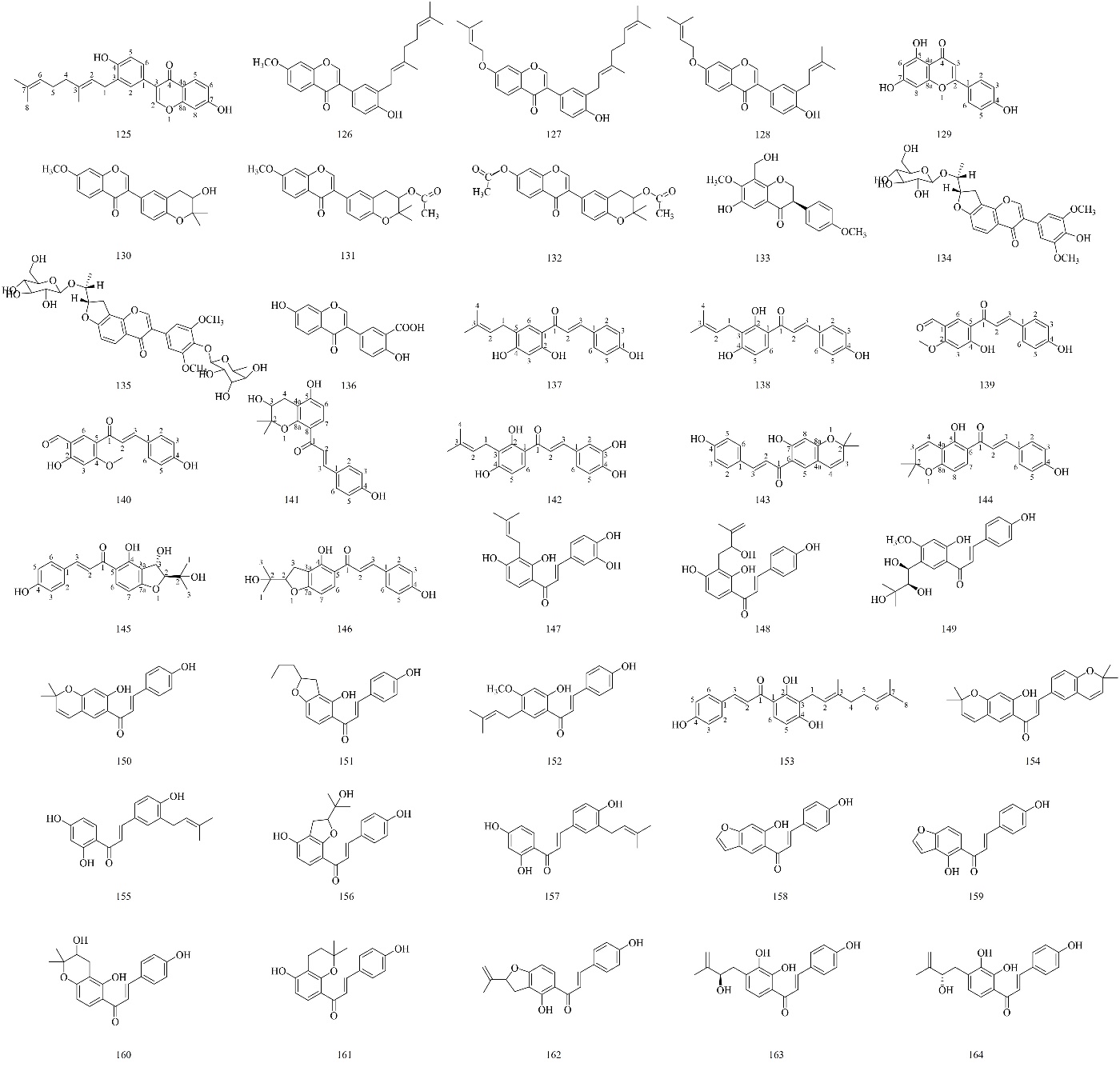 |
| (D) |
| 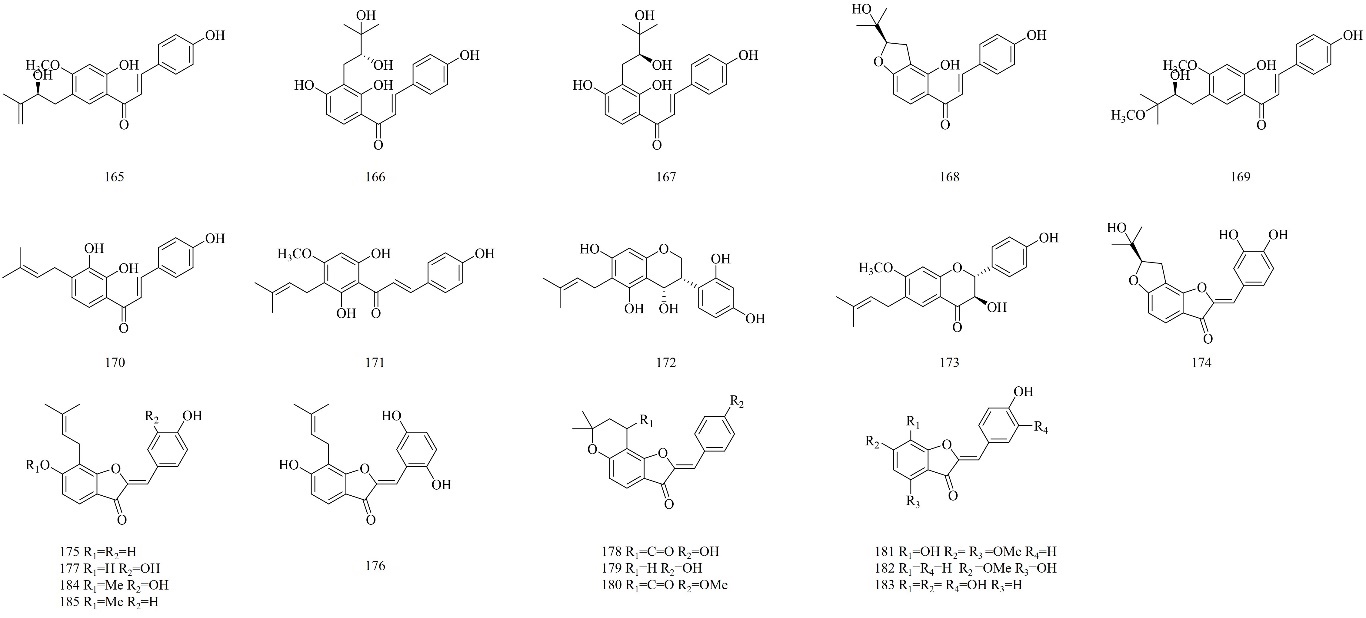 |
| (E) |
| 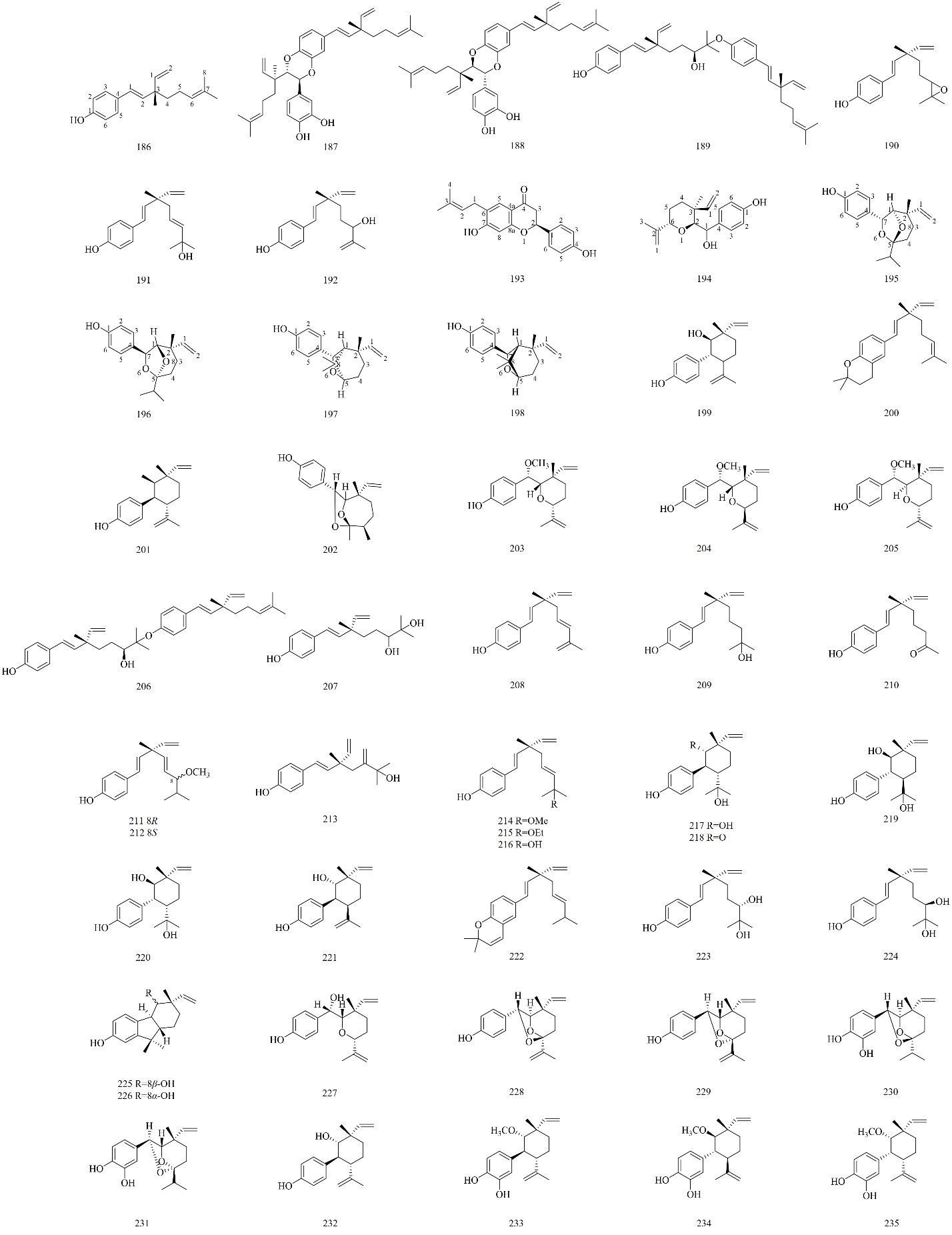 |
| (F) |
| 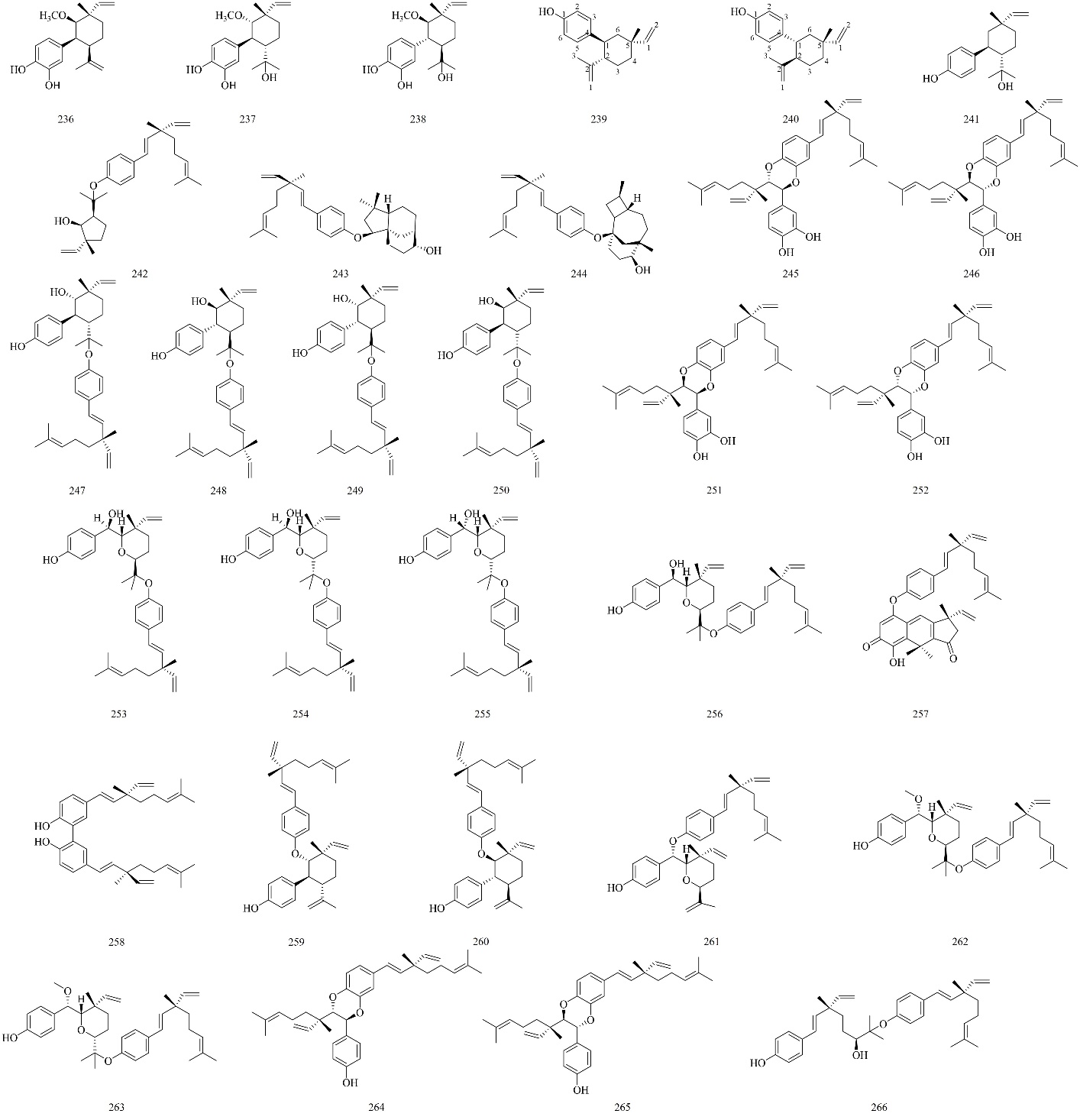 |
| (G) |
| 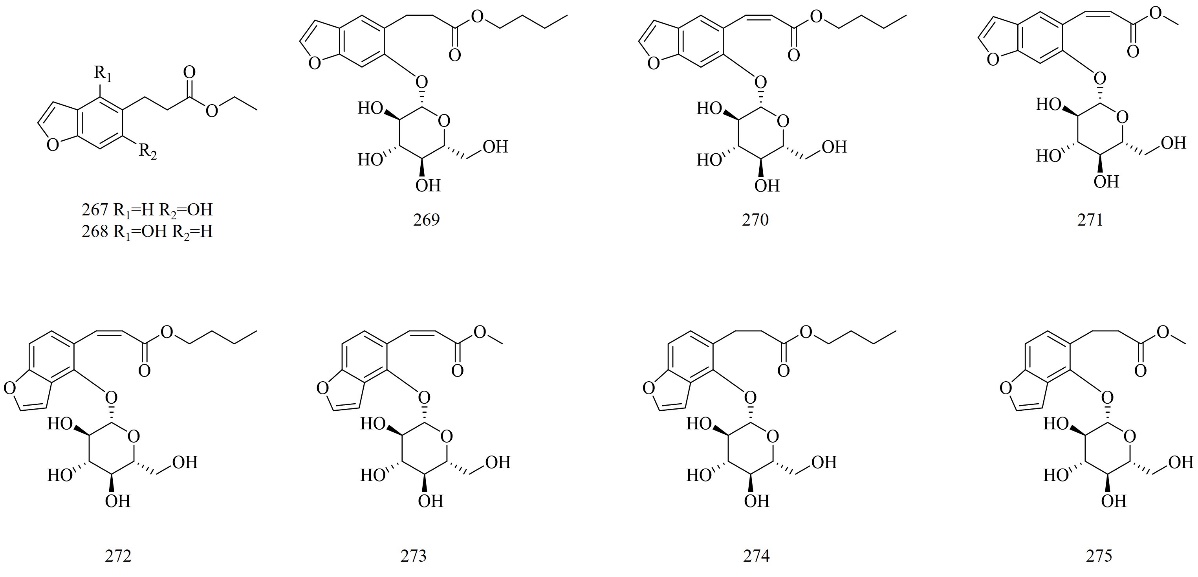 |
| (H) |
| 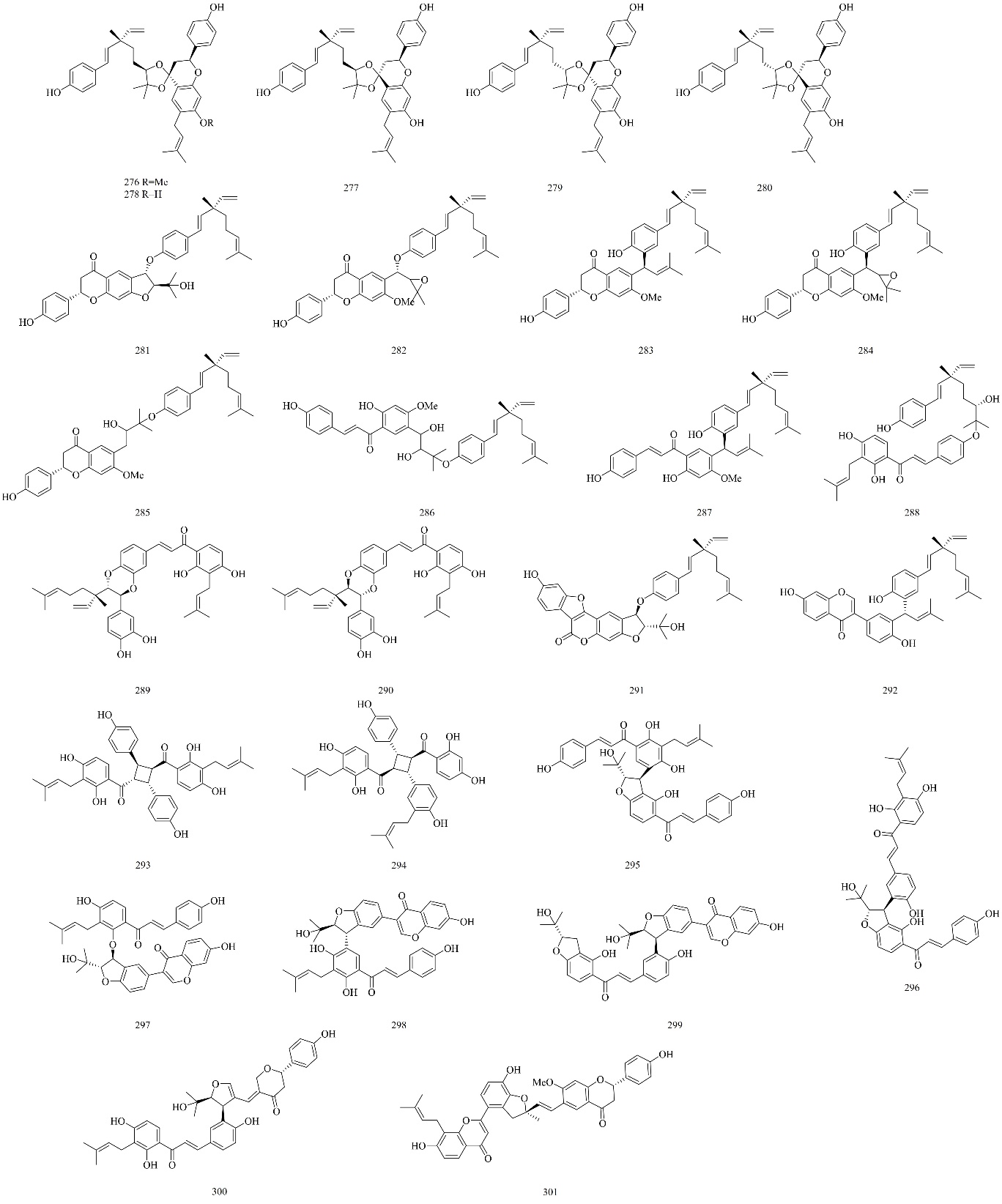 |
| (I) |
| 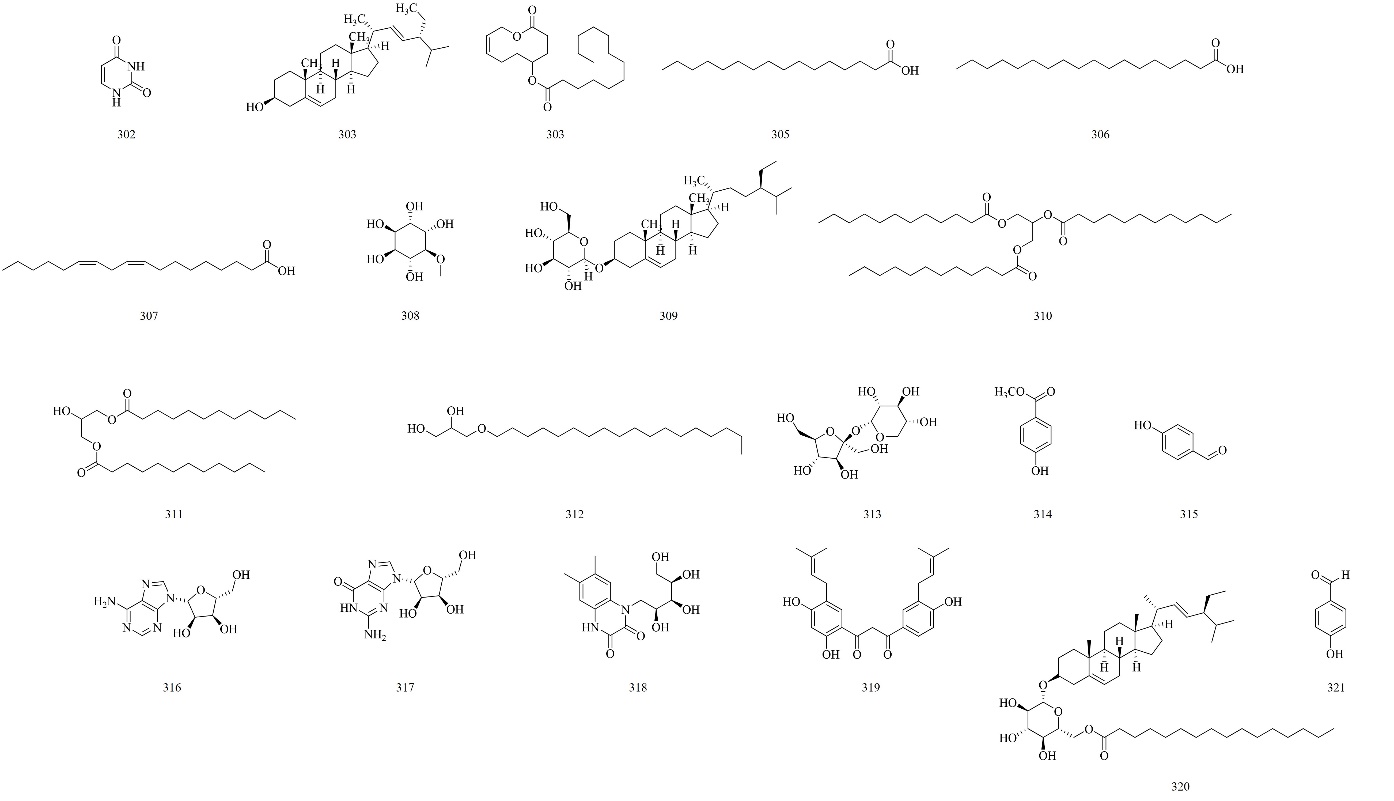 |
| (J) |

**Fig. S2** Coumarins (A), Flavonoids (B-E), Meroterpenes (F-G), Benzofurans (H), Dimers (I) and other metabolites (J) in *Psoralea corylifolia* L.

| 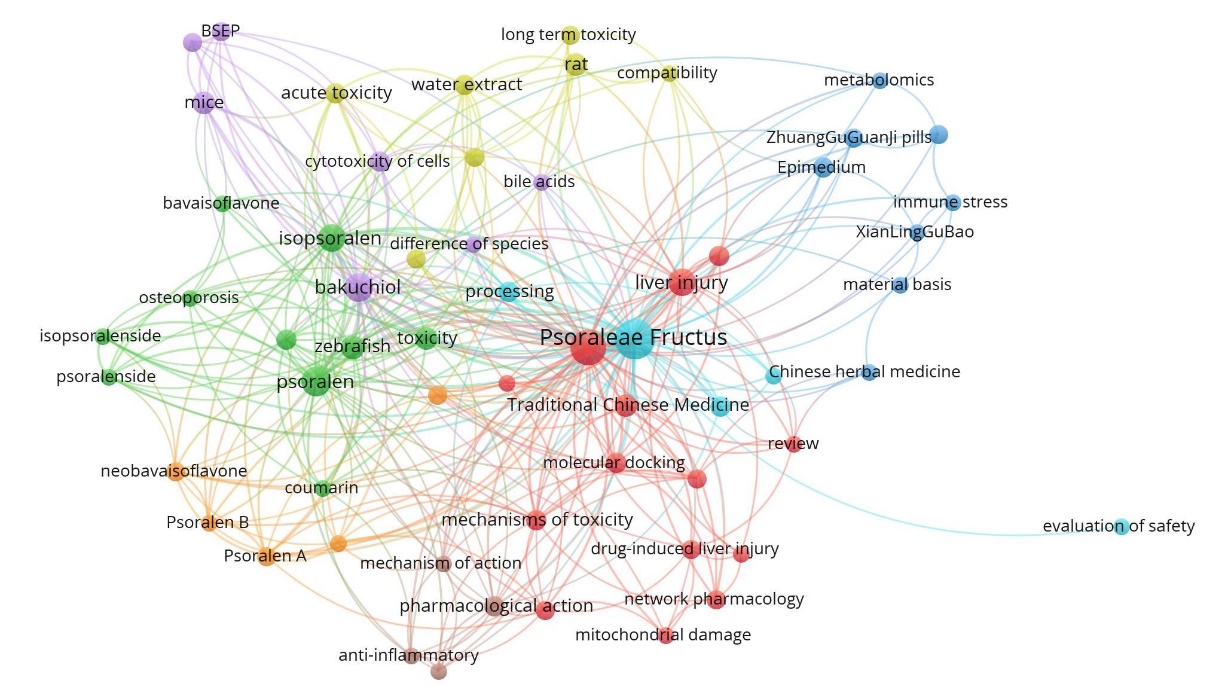 |
| --- |
| (A) |
| 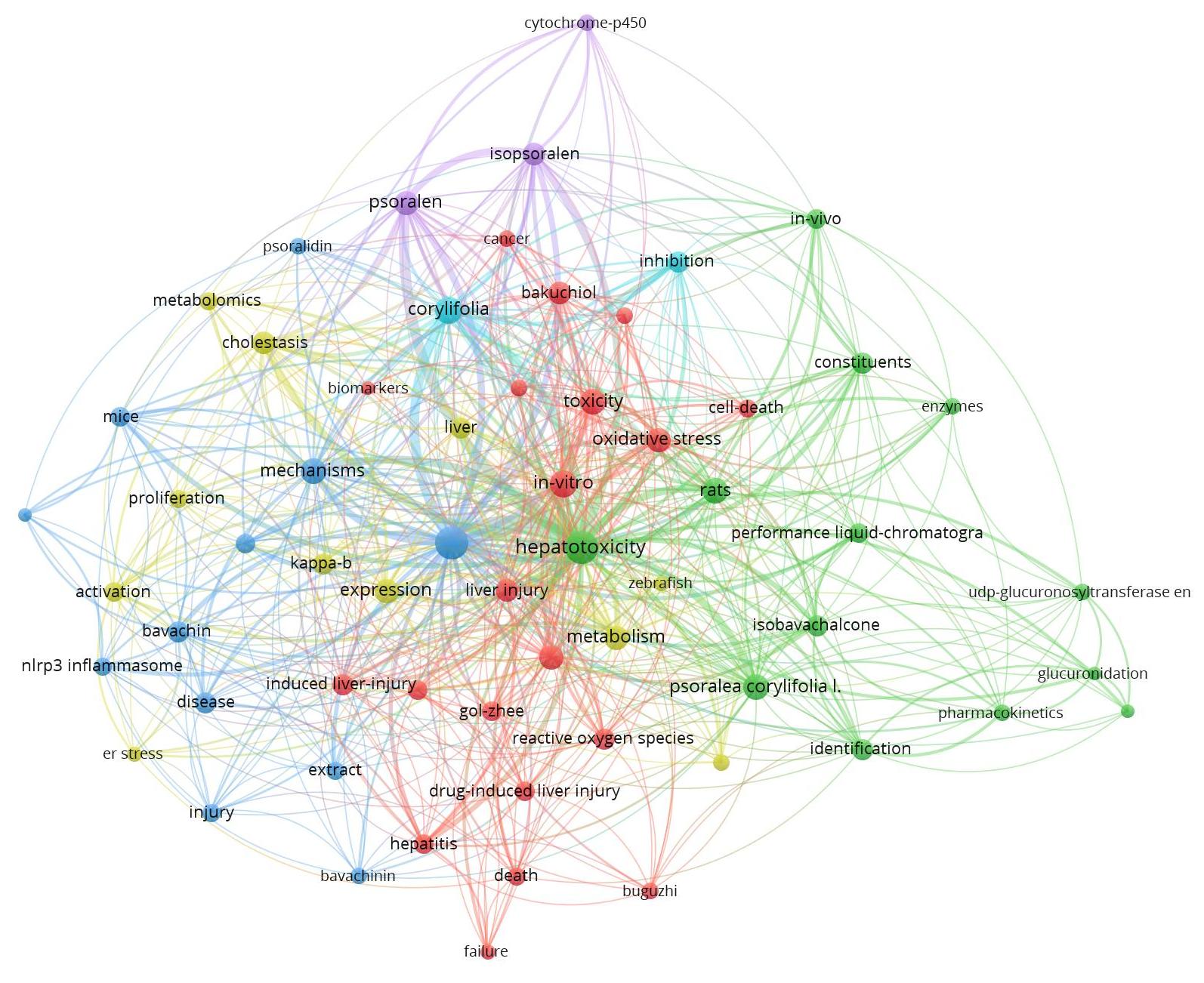 |
| (B) |

**Fig. S3** Co-occurrence atlas of Chinese keywords (A) and English keywords (B) in the Research Field of Liver Injury of *Psoralea corylifolia* L.

Abbreviations

| AD | Alzheimer's disease |
| --- | --- |
| AhR | Aryl hydrocarbon receptor |
| AKT | Protein kinase B |
| ALP | Alkaline phosphatase |
| ALT | Alanine aminotransferase |
| APP | Amyloid precursor protein |
| AQP | Aquaporin |
| AST | Aspartate aminotransferase |
| BGP | bone-γ-carboxyglutamic acidcontaining protein |
| BMD | Bone mineral density |
| BMP4 | bone morphogenetic protein-4 |
| BSEP | Bile Salt Export Pump |
| cAMP | Cyclic adenosine monophosphate |
| cGMP | Cyclic guanosinc monophosphate |
| DN | Diabetic nephropathy |
| E2 | Estradiol |
| EMT | Epithelial-mesenchymal transition |
| ER | Estrogen receptor |
| ERK | Extracellular regulated protein kinases |
| ERα | Estrogen receptor α |
| ERβ | Estrogen receptor β |
| FXR | Farnesoid X Receptor |
| GAS5 | Growth arrest-specific 5 |
| GSH | Glutathione |
| GSK-3β | Glycogen synthase kinase-3 beta |
| HCC | Hepatocellular carcinoma |
| HO-1 | Heme oxygenase 1 |
| JAK | Janus Kinase |
| JNK | C-jun N-terminal kinase |
| LPS | Lipopolysaccharide |
| MAPK | Mitogen-activated protein kinase |
| MCF-7 | Human breast cancer cell line |
| MDA | Malondialdehyde |
| MFN2 | Mitochondrial fusion protein 2 |
| MIC | Minimum Inhibitory Concentration |
| NF-κB | NF-κB |
| NO | Nitric oxide |
| Nrf2 | Nuclear factor erythroid 2-related factor 2 |
| NTCP | Sodium taurocholate cotransporting polypeptide |
| OC | Osteocalcin |
| OP | Osteoporosis |
| PCL | *Psoralea corylifolia* L. |
| PCNA | Proliferating Cell Nuclear Antigen |
| PF | Psoraleae Fructus |
| PI3K | Phosphatidylinositide 3-kinases |
| PPAR-γ | Peroxisome proliferators activate receptors-γ |
| RANK | Receptor activator of nuclear factor-κB |
| RANKL | Receptor activator of nuclear factor-κB ligand |
| ROS | Reactive oxygen species |
| SIRT1 | Silent information regulator 1 |
| SOD | Superoxide dismutase |
| STAT | Signal transducer and activator of transcription |
| SULT1E1 | Sulfotransferase family 1E |
| TCM | Traditional Chinese medicine |
| TNF-α | Tumor necrosis factor α |
| TRAP | Triiodothyronine receptor auxiliary protein |
| TRPC3 | Transient receptor potential channel |
| VEGF | Vascular endothelial growth factor |
